# Radical-Mediated C3-Alkylation of Coumarins: A Comprehensive Review

**DOI:** 10.1021/acsomega.6c03533

**Published:** 2026-06-17

**Authors:** Clarice Alves Dale Caiuby, Arthur de Sousa Vasconcelos, Marco Antonio Barbosa Ferreira

**Affiliations:** † Institute of Education Sciences (ICED), Federal University of Western Pará (UFOPA), Rua Vera Paz, s/n, Salé, Santarém, Pará 68040-255, Brazil; ‡ Chemistry Department, Federal University of São Carlos (UFSCar), Rodovia Washington Luís, km 235—SP—310, São Carlos, São Paulo 13565-905, Brazil

## Abstract

Radical chemistry has emerged as a powerful platform for the construction of carbon–carbon and carbon–heteroatom bonds under mild and operationally simple conditions. In this context, the direct functionalization of heterocyclic scaffolds has attracted significant attention because of its potential for rapid molecular diversification and late-stage modification of bioactive compounds. Among these scaffolds, coumarins occupy a prominent position owing to their widespread occurrence in natural products, pharmaceuticals, and functional materials. Although classical approaches have enabled efficient access to coumarin cores, the direct and selective functionalization of the C3 position remains a significant challenge. In recent years, radical-based methodologies have become powerful and versatile strategies for overcoming this limitation, allowing efficient C3-alkylation of coumarins without the requirement for prefunctionalized substrates. This review provides a comprehensive overview of recent advances in the radical-mediated C3-alkylation of coumarins. The discussion is organized according to the activation mode, highlighting key developments in thermal and photoredox strategies. Emphasis is placed on mechanistic aspects, substrate scope, and synthetic applicability, offering a critical perspective on the advantages and limitations associated with each approach. Finally, emerging trends and future opportunities for the development of more efficient, selective, and sustainable methodologies for C3-functionalization of coumarins are discussed.

## Graphical abstract

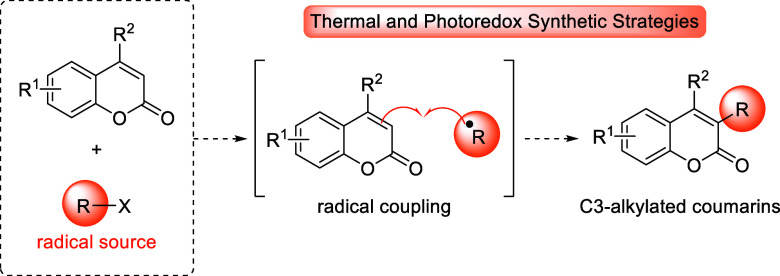

## Introduction

Radicals are open-shell reactive intermediates of great importance in modern synthetic chemistry. For a long time, inherent reactivity and short lifetimes of carbon-centered radicals were considered a major limitation to achieving selective transformations. However, the past few decades have witnessed substantial progress in the development of strategies that enable the controlled generation and utilization of radical species.^1–5^ Advances in catalyst design and reaction methodologies now allow radicals to be formed under mild and well-defined conditions, greatly expanding their synthetic potential.^6–20^ Experimental techniques, such as electron paramagnetic resonance (EPR) spectroscopy,^21,22^ combined with kinetic studies and computational methods,^23–30^ have provided valuable insights into the nature of radical intermediates and their reaction pathways. This deeper understanding has allowed chemists to rationally design reaction systems that promote the desired transformations while minimizing competing processes, thus establishing radical chemistry as a powerful platform for the construction of new carbon–carbon and carbon–heteroatom bonds. Specifically, direct radical-mediated alkylation of heterocycles has emerged as a powerful and widely used strategy in synthetic chemistry.^31–34^ It enables the rapid construction of molecular complexity from simple, readily available feedstock chemicals, making it particularly attractive for the late-stage functionalization of heterocyclic structures commonly found in biologically active compounds and pharmaceuticals.

Coumarins are privileged structural motifs in organic chemistry. Frequently found in natural products,^35–39^ biologically relevant compounds,^40–44^ and fluorescent materials,^45–51^ this class of compounds exhibits a broad range of applications across medicinal chemistry, materials science, and photophysics. Owing to their remarkable structural diversity and functional versatility, both the *novo synthesis* and selective functionalization of coumarins have been extensively investigated over the past decades.^52–56^

Traditional synthetic approaches for coumarin synthesis typically rely on well-established condensation methodologies, including the Pechmann,^57–59^ Knoevenagel,^60–63^ Perkin,^64–66^ Wittig,^67–69^ and Reformatsky^70–72^ reactions (Scheme 1A). However, these strategies often require multiple synthetic steps, extended reaction times, and the use of prefunctionalized substrates that are not always readily available. In contrast, direct C–H functionalization has emerged as an atom- and step-economical alternative for the rapid diversification of coumarin scaffolds (Scheme 1B).^73–75^ In particular, the C4 position has been previously explored due to its intrinsic electrophilicity, which facilitates various transformations, such as nucleophilic conjugate-type or metal-catalyzed reactions under mild conditions.^76–79^

**Scheme 1:**
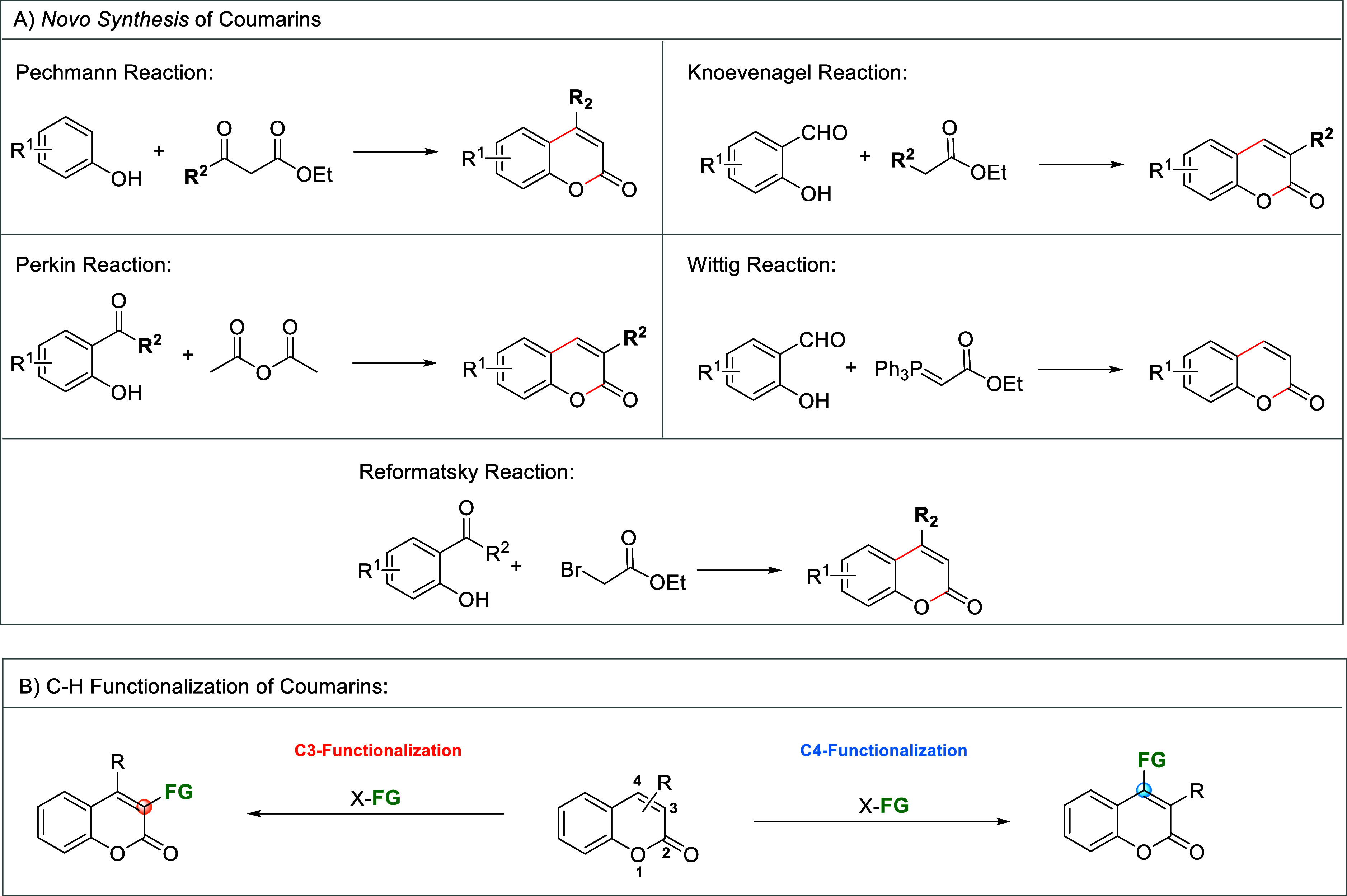
Synthesis and Functionalization of Coumarins

Among coumarin derivatives, C3-alkyl-substituted analogues are particularly noteworthy due to their significant pharmacological activities.^44,80–85^ This scaffold is featured in several commercially available anticoagulant drugs,^86–89^ including Warfarin, Phenprocoumon, Acenocoumarol, and Dicoumarol (Figure 1). Beyond anticoagulant activity, C3-alkyl substitution in coumarins has been associated to anticancer,^90–93^ antimicrobial,^94–97^ and antiviral properties,^98,99^ further highlighting the value of this class of compounds. Despite their significant biological relevance, the development of efficient and selective methods for direct C3-functionalization of coumarins remains a considerable synthetic challenge, especially when avoiding prefunctionalized substrates.

**Figure 1:**
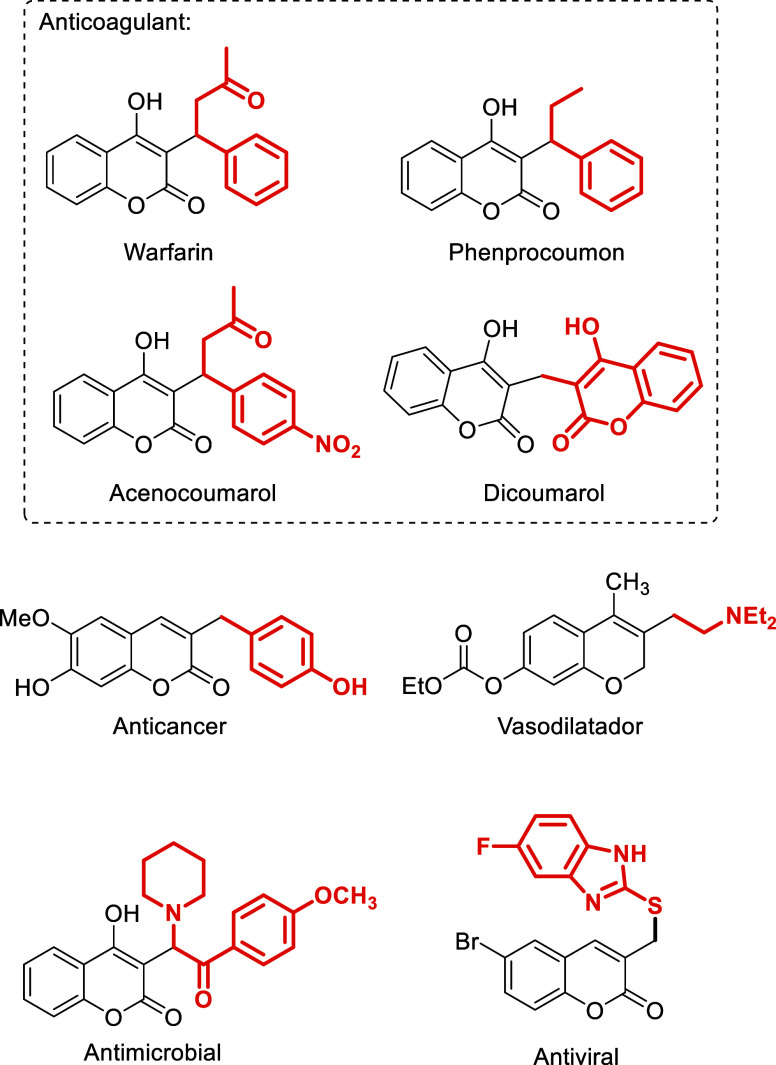
Examples of bioactive C3 functionalized coumarins.

In this context, radical-based transformations are highly attractive, as they usually proceed under mild conditions and exhibit broad functional group tolerance, thereby enabling efficient and direct access to structurally diverse coumarin derivatives. To this end, alkyl radicals can be generated from a variety of precursors, including alkyl halides, oxime esters, alkyl amines, boronic acids, carboxylic acids, redox-active esters, alkylbenzenes, diacyl peroxides, alkyl ethers, and Katritzky salts. The general mechanism for site-selective radical addition at the C3 position of coumarins leads to the formation of a stabilized benzylic radical intermediate, which plays a pivotal role in the construction of the final alkylated products (Scheme 2). This review provides a comprehensive overview of the main advances in C3-radical alkylation of coumarins, categorizing reported methodologies according to the main reaction activation mode: thermal and photoredox reaction conditions. Particular emphasis is placed on mechanistic insights, substrate scope, and synthetic applicability, aiming to offer a clear perspective on current achievements and future opportunities in this rapidly evolving field. Methodologies focused on the exclusively fluoroalkylation of coumarins will not be discussed in this review, which will instead focus on simple alkylation strategies.

**Scheme 2:**
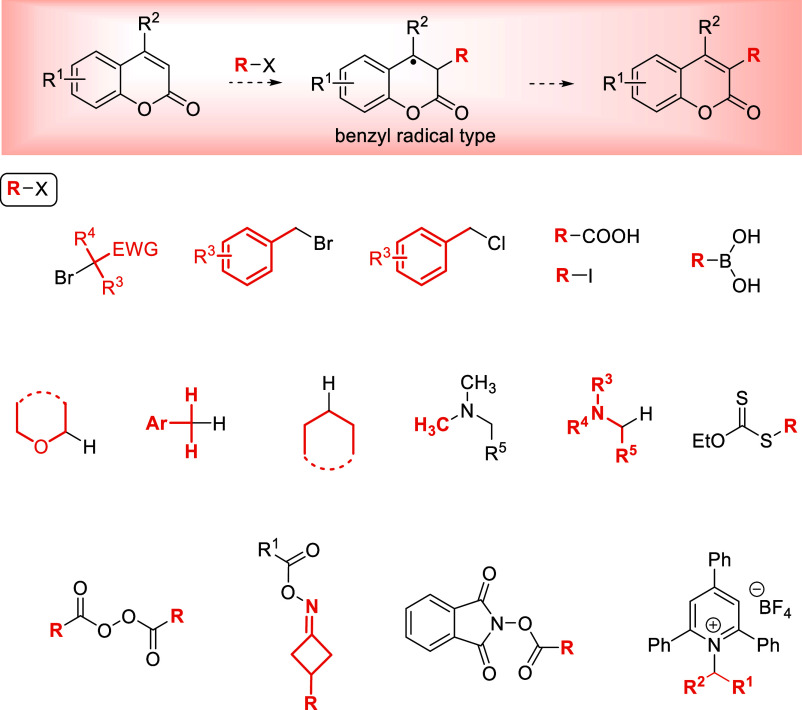
Radical Sources for C3-Alkylations of Coumarins

## Thermal Reaction Conditions

### Alkyl Halides

Exploiting the inherent lability of the C–Br bond in activated alkyl bromides during studies on the direct alkylation of heteroarenes, Miura and Evano independently reported the selective C3-alkylation of coumarins catalyzed by nickel and copper complexes, respectively (Scheme 3A,B).^100,101^ A few years later, Shen and Li developed an iron-catalyzed approach for the regioselective radical alkylation of quinones and coumarins using functionalized alkyl bromides (BrCH_2_-EWG and benzyl bromides) as alkylating reagents (Scheme 3C).^102^ In this later work, the reaction conditions were initially optimized using ethyl 2-bromopropanoate and 2-methylnaphthalene-1,4-dione as model substrates, but subsequently extended to coumarin derivatives. Optimization experiments revealed that the combination of FeCl_2_ (7.5 mol %) and DIPEA (2.0 equiv) in toluene at 130 °C for 45 h efficiently promoted the transformation, affording the desired C3-alkylated coumarin products in moderate to good yields (27–71%). Notably, the transformation did not exhibit a pronounced electronic effect, accommodating both electron-donating and electron-withdrawing substituents on the coumarin scaffold. Control experiments confirmed that both the iron catalyst and the base were essential for the reaction, as no product formation was observed in their absence. Mechanistic investigations, including radical-trapping experiments, support a radical pathway. The authors proposed a catalytic cycle initiated by single-electron transfer (SET) from an Fe­(II) species to the alkyl bromide, generating an alkyl radical along with an Fe­(III) species. The resulting radical subsequently adds to the C3 position of the coumarin substrate, forming a benzylic radical intermediate. This intermediate then undergoes oxidation, followed by proton elimination, to regenerate the Fe­(II) catalyst and furnish the final alkylated product.

**Scheme 3:**
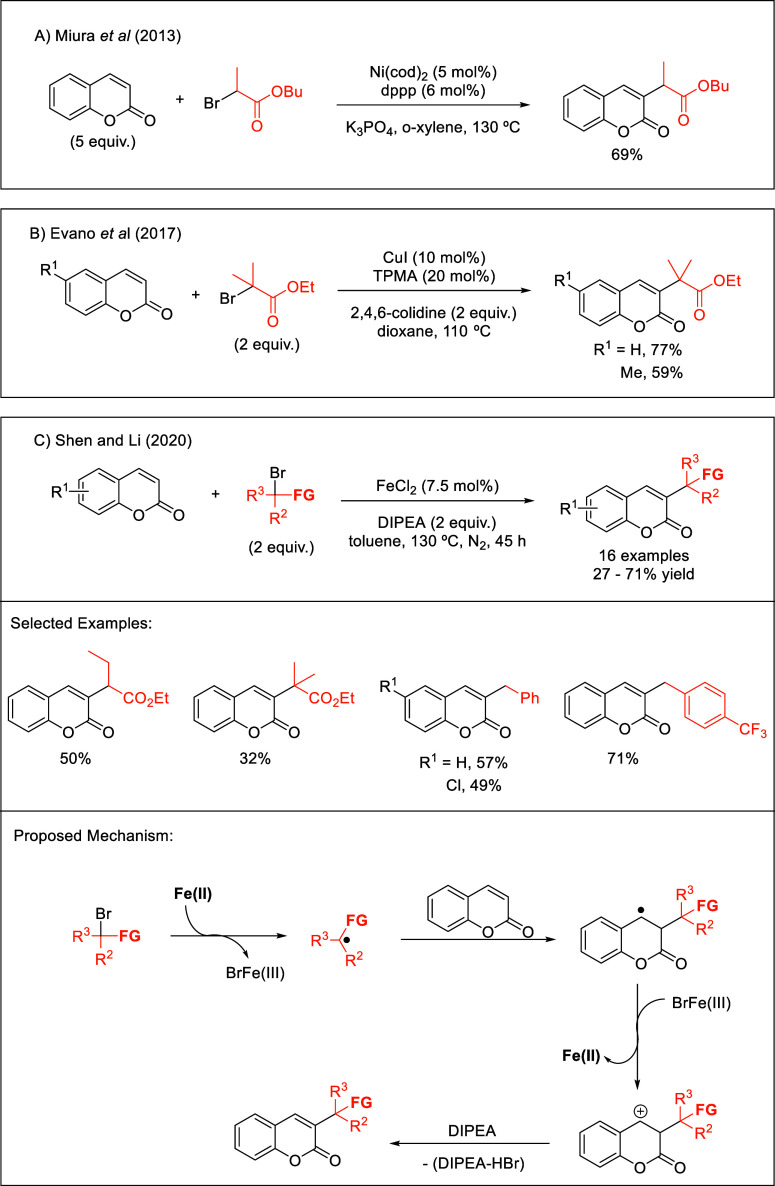
Activated Alkyl Bromides in Metal-Catalyzed Alkylation of Coumarins

Concurrently, Yang and Li also reported the use of activated alkyl halides, such as benzyl chlorides, benzyl bromides, and 2-bromoacetonitrile, as radical precursors for the copper-catalyzed radical-alkylation of coumarins (Scheme 4).^103^ The transformation was promoted by a Cu­(II)/phenanthroline catalytic system under basic conditions at 130 °C, affording the desired product in 88% yield after 28 h. The substrate scope revealed good tolerance toward different substitution patterns on the coumarin framework. Both electron-donating and electron-withdrawing substituents were well tolerated, and coumarins bearing methyl, methoxy, halogen, and amino groups furnished the corresponding C3-alkylated products in good to excellent yields (61–91%). When the method was applied using a C4-methyl-substituted coumarin, the desired product was obtained in 39% yield after 48 h. Evaluation of the alkyl halide scope demonstrated that a variety of primary alkyl halides (X = Br, Cl) could be successfully employed, affording the desired products in moderate to excellent yields (32–92%). In contrast, the use of secondary alkyl bromides proved less efficient, providing the corresponding C3-alkylated coumarin in only 32% yield. The synthetic utility of the obtained products was further demonstrated through subsequent benzylic oxidation and intramolecular C–H activation transformations. The proposed catalytic cycle for this transformation is initiated by single-electron transfer (SET) from the copper catalyst to the alkyl halide, generating an alkyl radical together with a Cu­(III) species. The resulting radical undergoes regioselective addition at the electron-deficient C3 position of the coumarin, producing a new carbon-centered radical intermediate. This intermediate is subsequently oxidized, likely by the copper catalyst, to generate a carbocation species. Finally, deprotonation restores aromaticity to afford the C3-alkylated coumarin while regenerating the active copper catalyst, thus completing the catalytic cycle. The involvement of radical intermediates was supported by radical scavenger experiments.

**Scheme 4:**
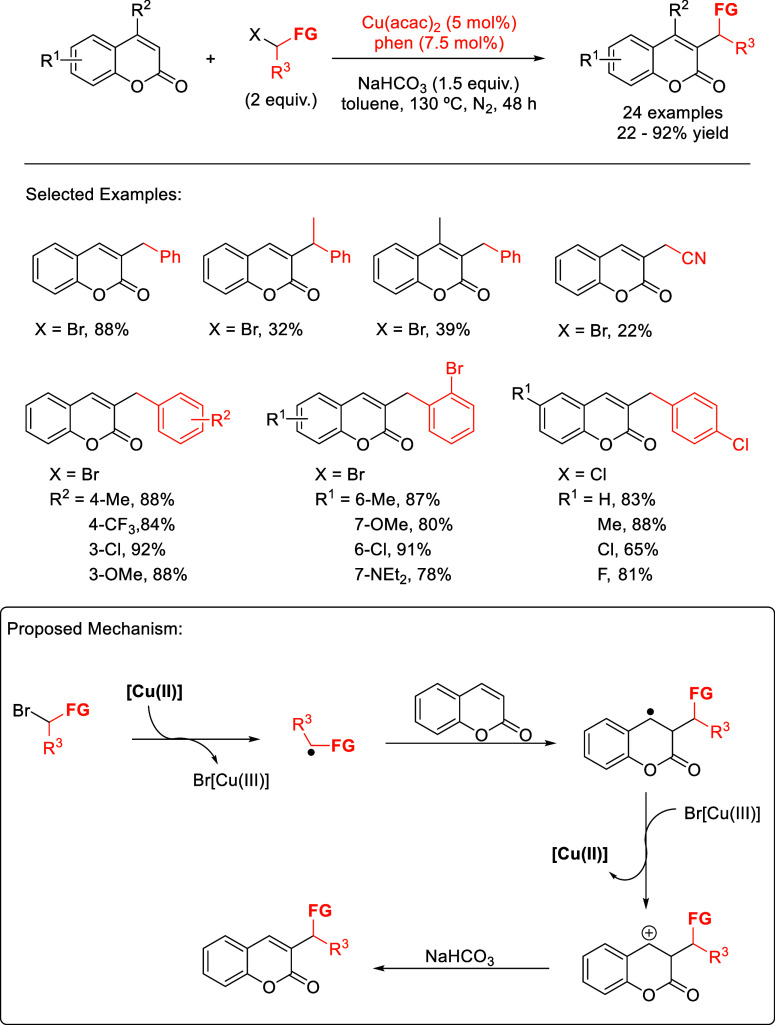
Copper-Catalyzed Radical Alkylation of Coumarins Using Alkyl Halides

Overall, despite successfully exploiting earth-abundant metals for these transformations, notable drawbacks of these strategies include the requirement for high reaction temperatures and their reliance on activated alkyl halides. Although the electronic activation of these substrates ensures high reactivity, it simultaneously narrows the substrate scope, highlighting the need for methods capable of engaging unactivated counterparts.

### C–H Bond of Alkanes, Ethers, and Amines

The C–H bonds of alkanes represent abundant and valuable precursors for generating alkyl radicals (R^•^) via homolytic bond cleavage. This approach bypasses the need for prefunctionalized alkyl halides and avoids stoichiometric halogen waste, thereby inherently maximizing atom economy. However, owing to the high bond dissociation energies (BDEs) and lack of polarity of these bonds, their direct activation generally requires an efficient radical initiation step. Among the strategies developed to address this challenge, the decomposition of peroxides remains a classical and widely used approach.^104–106^ In this process, peroxide-derived radicals abstract a hydrogen atom from the alkane via a hydrogen atom transfer (HAT) mechanism. This generates the corresponding alkyl radical which can subsequently engage in a variety of C–C and C–X bond-forming reactions. In 2014, Wei and Zhu applied this strategy to promote a Cu-catalyzed C–H coupling of unfunctionalized hydrocarbons with styrenes or heteroaromatic compounds (Scheme 5).^107^ The transformation relies on the metal-mediated decomposition of *tert*-butyl peroxide (TBP), which generates a *tert*-butoxy radical capable of abstracting a hydrogen atom from the alkane to form the corresponding alkyl radical. Subsequent addition of this alkyl radical to the double bond produces a stabilized benzylic radical intermediate, which is proposed to undergo oxidation followed by deprotonation to furnish the final product while regenerating the active copper catalyst. The authors demonstrated the feasibility of this approach for the C3-alkylation of coumarins, reporting two representative examples utilizing cyclohexane as the radical precursor, which furnished the desired products in 70–73% yield.

**Scheme 5:**
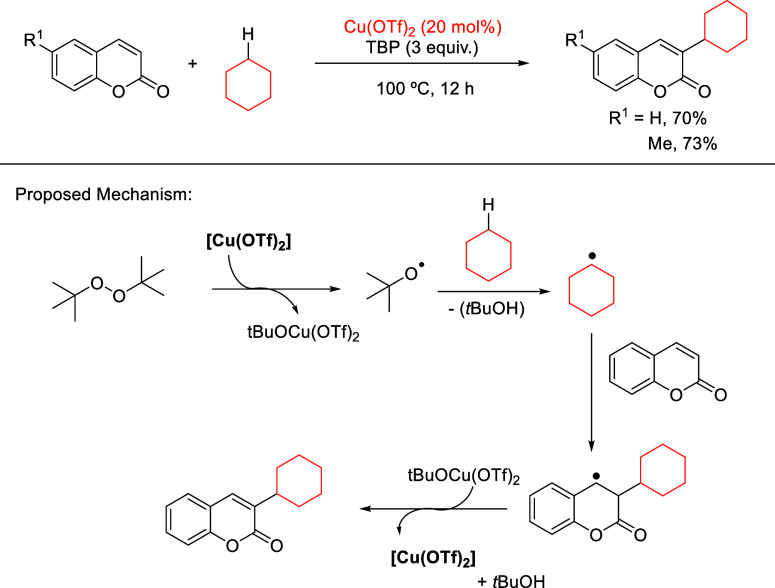
Copper-Catalyzed Radical Alkylation of Coumarin Using Cyclohexane

Accessing alkyl radicals from simple alkanes remains a significant challenge due to their low reactivity and the difficulty in achieving regioselectivity in more complex structures, thereby severely limiting the substrate scope. Consequently, this strategy can be applied with greater efficiency by utilizing C–H bonds adjacent to activating functional groups. In 2014, Duan and co-workers developed a similar approach enabling the direct benzylation of coumarins via cross-dehydrogenative coupling (Scheme 6).^108^ Reaction optimization revealed that copper­(II) acetate [Cu­(OAc)_2_] combined with *tert*-butyl peroxybenzoate (TBPB) as the oxidant provided the best results. Under these optimized conditions, the reaction of coumarin with toluene at 100 °C for 24 h produced the corresponding 3-benzylcoumarin in 80% yield. A variety of *para*- and *ortho*-substituted toluene derivatives were successfully employed as radical sources, producing the corresponding benzylated coumarins with good regioselectivity in 60–80% yield. Functional groups such as halogens, acetyl, and cyano substituents were well tolerated under the reaction conditions. Notably, toluene derivatives containing multiple methyl groups resulted exclusively the monobenzylated products in 62–80% yield. Additionally, substituted coumarins bearing electron-donating or electron-withdrawing groups on the aromatic ring were also compatible with the reaction. In general, coumarins bearing electron-donating groups showed higher reactivity and gave better yields (72–86%), whereas electron-withdrawing substituents led to lower conversions (27–46%), suggesting that the electronic effects of the substituents on the phenyl ring exert a significant effect on the reactivity of the coumarins. The methodology was further extended to other Csp^3^-H substrates beyond simple benzylic hydrocarbons, such as secondary alkanes, alcohols, and ethers, although with lower efficiency (up to 39% yield).

**Scheme 6:**
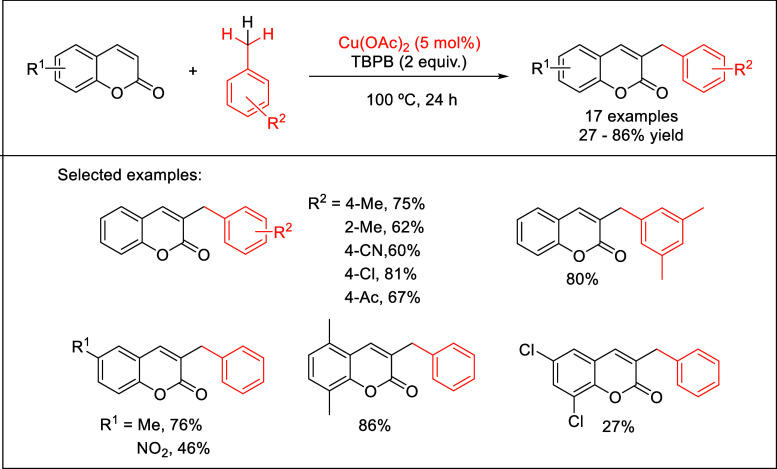
Copper-Catalyzed Radical Alkylation of Coumarin Using Toluene Derivatives

Functional group-activated C–H bonds can also be exploited as radical precursors in transition-metal-free protocols. A representative example includes the regioselective C3-cyanomethylation and acetomethylation of coumarins developed by Yan and co-workers. This transformation is promoted by TBPB (2.5 equiv) as a radical initiator in the presence of KF (1.0 equiv) under neat conditions (Scheme 7).^109^ Using this protocol, the cyanomethylation of coumarins bearing a variety of substituents, such as Me, OMe, OEt, and NEt_2_, proceeded smoothly, affording the desired products in moderate to good yields (58–78%). The methodology also proved compatible with more structurally complex substrates, including benzocoumarins and furocoumarins, which were converted to the corresponding functionalized products in 56–83% yield. Notably, substitution at the C4 position of the coumarin scaffold was well tolerated, as evidenced across several examples. The authors further demonstrated the versatility of this approach by extending it to the 3-acetomethylation of coumarins under analogous conditions, furnishing a series of acetomethylated derivatives in moderate to good yields (48–74%). The transformation is proposed to proceed through a radical pathway initiated by the thermal homolysis of TBPB, generating *tert*-butoxy and benzoyloxy radicals. These radical species promote hydrogen-atom abstraction from the functionalized alkane, leading to formation of the corresponding alkyl radical. Subsequent radical addition to the electron-deficient C=C bond of the coumarin furnishes a benzyl radical intermediate. Oxidation of this intermediate by either *tert*-butoxy or benzoyloxy radicals, followed by deprotonation, finally delivers the C3-functionalized product. Experimental observations strongly support the involvement of a radical process. The reaction was completely suppressed in the presence of the radical scavengers TEMPO or BHT. Moreover, GC–MS analysis revealed the formation of the BHT-CH_2_CN adduct, providing direct evidence for the generation of a cyanomethyl radical intermediate under the reaction conditions.

**Scheme 7:**
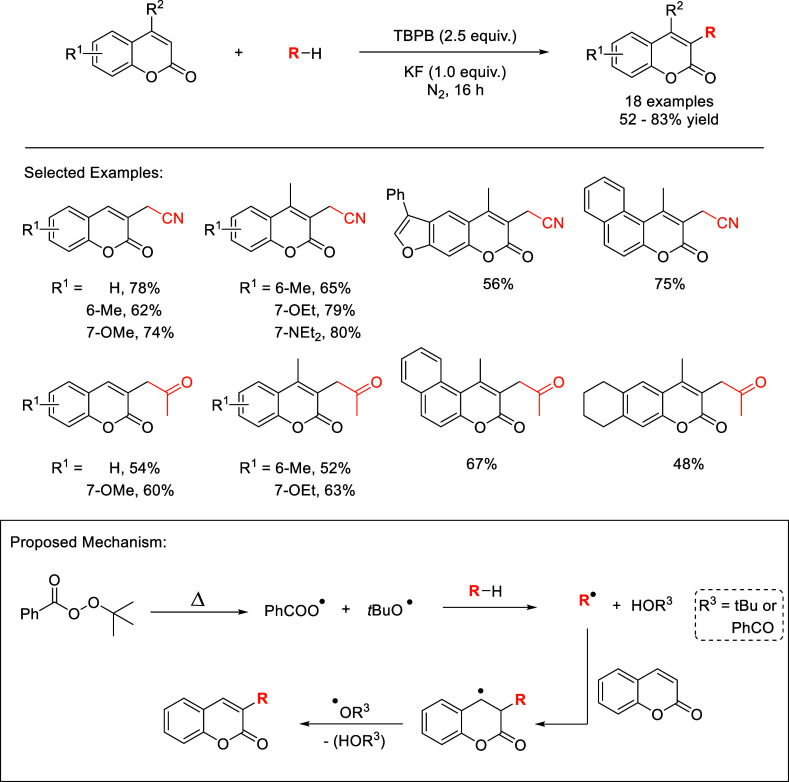
Metal-Free Radical Cyanomethylation and Acetomethylation of Coumarins

The α-C–H bonds of ethers can also undergo oxidative activation in the presence of peroxides to generate α-heteroatom-carbon-centered radicals. Independent studies by the research groups of Ge, Wu, and Du employed this strategy in cross-dehydrogenative coupling reactions catalyzed by iron, copper, and cobalt complexes, respectively (Scheme 8A–C).^110–112^ These methodologies proved highly effective for the C3 radical alkylation of coumarins using a broad range of cyclic and acyclic ethers, providing the desired products in up to 95% yield. In general, coumarins bearing electron-donating groups were well tolerated under the reaction conditions. In contrast, substrates containing strongly electron-withdrawing substituents, such as nitro group, failed to deliver the corresponding alkylated products.

**Scheme 8:**
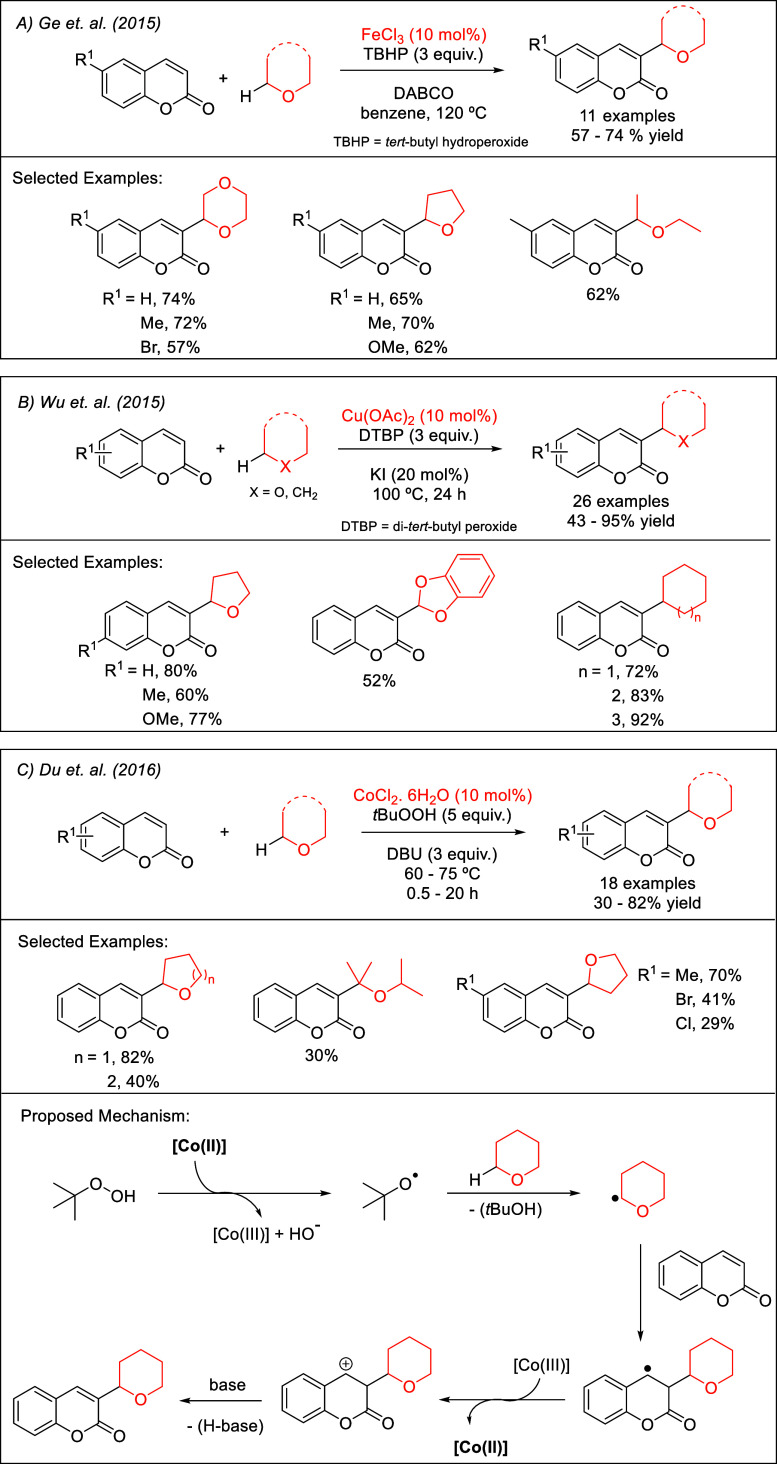
Metal-Catalyzed C3-Alkylation of Coumarins Using Ethers and Cycloalkanes

Additionally, in the study reported by Wu and co-workers, replacement of Cu­(OAc)_2_ with Cu­(OTf)_2_ enabled the efficient direct alkylation of coumarins using simple alkanes, without the need for additional additives (Scheme 8B). In all cases, the radical nature of the transformation was supported by appropriate radical-trapping experiments. Although the individual systems present specific features, the proposed mechanisms follow a common pathway as exemplified in Scheme 8C. Initially, metal-promoted homolytic cleavage of the peroxide generates a reactive radical species capable of abstracting a hydrogen atom from the ether to produce an α-oxyalkyl radical. Subsequent addition of this radical to the C3 position of the coumarin affords a benzylic radical intermediate, which undergoes oxidation followed by deprotonation to furnish the final product with concomitant regeneration of the catalytic species.

In 2018, Darvishmolla and Jafarpour described a peroxide-mediated cross-dehydrogenative coupling strategy for the direct C3 alkylation of coumarins under transition-metal-free conditions (Scheme 9).^113^ This protocol utilizes *tert*-butyl hydroperoxide (TBHP) as the sole oxidant to promote the coupling between coumarins and unactivated ethers via C–C bond formation. Remarkably, the reaction proceeds efficiently in the absence of catalysts, additives, and solvents. Under the optimized conditions (TBHP, 120 °C, 24 h), a variety of coumarin derivatives underwent selective alkylation at the C3 position, affording the desired products in moderate to good yields (45–78%). However, strongly electron-withdrawing groups, such as nitro substituents, led to no product formation. Additionally, coumarins bearing substitution at the C4 position afforded only trace amounts of the desired product, likely due to steric hindrance. A wide range of ethers, both cyclic and acyclic, participated effectively in the reaction as alkyl radical precursors. Simple alkanes, such as cyclohexane, were also viable substrates for this transformation, albeit with moderate efficiency (60% yield). Mechanistic investigations support a radical pathway. The inhibition of the reaction in the presence of TEMPO indicates the involvement of radical intermediates. The proposed mechanism involves the thermal homolysis of TBHP to generate *tert*-butoxy and hydroxyl radicals, which abstract hydrogen atoms from the ether substrate to form α-oxyalkyl radicals. These radicals subsequently add to the electron-deficient C3 position of the coumarin, generating a stabilized carbon-centered radical intermediate. Oxidation and deprotonation steps then furnish the final alkylated product. A particularly noteworthy extension of this methodology is its application to coumarin-3-carboxylic acids.

**Scheme 9:**
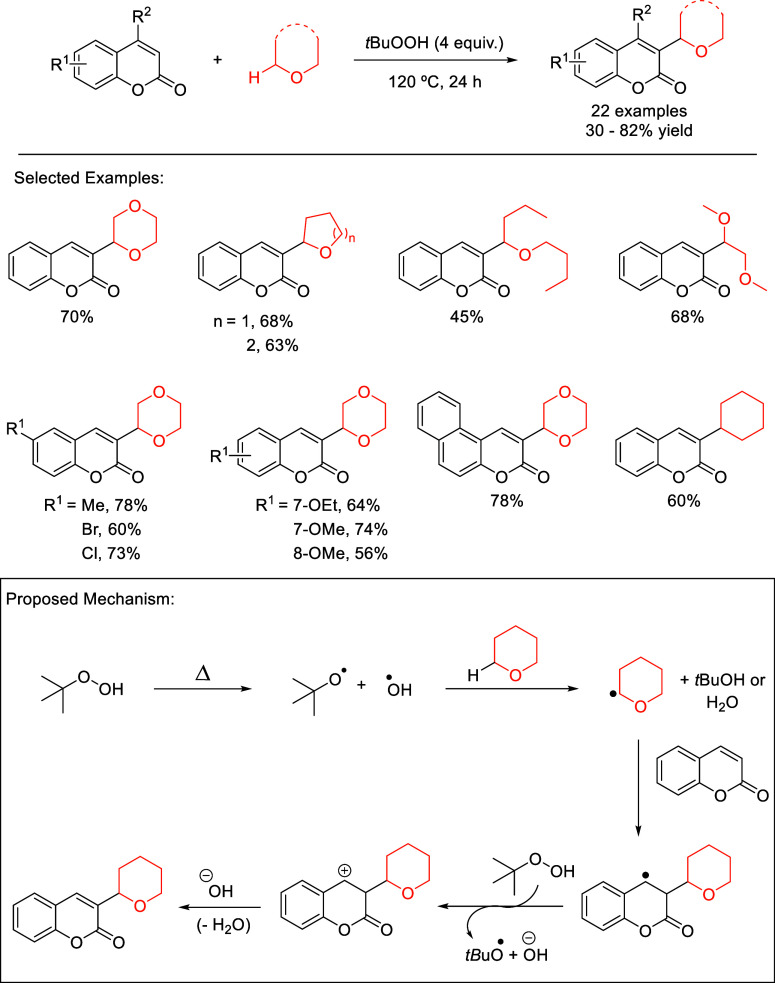
Metal-Free C3-Alkylation of Coumarins Using Ethers

Du and co-workers exploited the intrinsic reactivity of the α-C–H bonds of tertiary amines to develop a transition metal-free radical alkylation of coumarins (Scheme 10).^114^ The protocol relies on a combination of *n*Bu_4_NI and *t*BuOOH in the presence of DBU in acetonitrile at 75 °C. Under the optimized conditions, coumarin and an excess of *N,N*-dimethylaniline (3 equiv) were employed as model substrates, affording the desired C3-α-aminoalkylated product in 80% yield. Evaluation of the substrate scope revealed that coumarins bearing electron-donating substituents provided the corresponding products in moderate yields (45–60%), whereas halogen-substituted analogues afforded lower yields (25–37%). A coumarin bearing a methyl group at the C4 position delivered the desired product in 47% yield. Various tertiary amines were also examined, demonstrating tolerance toward alkyl, alkoxy, and halogen substituents (11–80% yield). A phenyl-substituted amine furnished the corresponding C3 alkylated product in 58% yield, while the diethyl-substituted analogue afforded only 20% yield due to incomplete conversion. Additionally, *N*-methyl-2-pyrrolidone, a commonly used organic solvent, could also undergo oxidation under the optimized conditions to generate the corresponding product, albeit in a modest yield of 11%. The proposed mechanism begins with the homolytic cleavage of *t*BuOOH, promoted by *n*-Bu_4_NI, generating *t*BuOO^•^ or t-BuO^•^ oxygen-centered radicals. Both species are capable of abstracting a hydrogen atom from the C–H bond of the methylene group adjacent to the nitrogen atom in tertiary amines, leading to the formation of an α-aminoalkyl radical. This radical subsequently adds to the electron-deficient C3 position of the coumarin, generating the relatively stable benzylic radical intermediate. Oxidation of this intermediate furnishes the corresponding cationic species, which then undergoes base-assisted deprotonation to deliver the final C3-alkylated product.

**Scheme 10:**
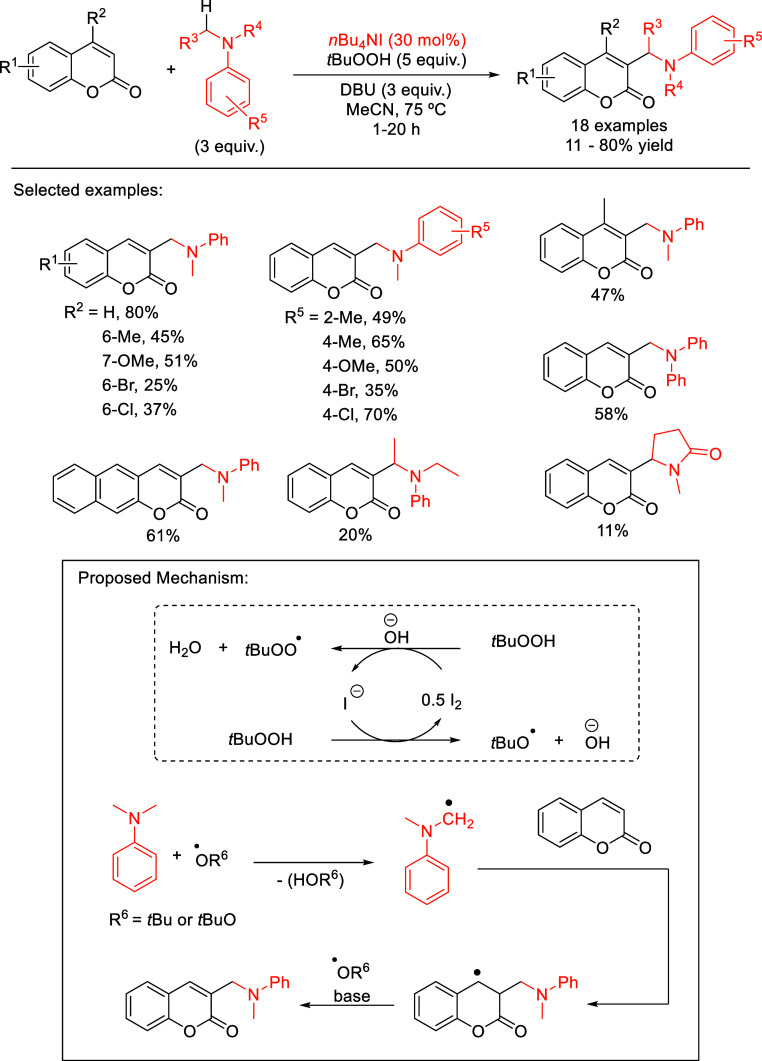
Radical C3-Alkylation of Coumarins Using Tertiary Amines

As evidenced by the aforementioned studies, the strategy of employing C–H bonds as radical precursors is fundamentally limited by the requirements of peroxide-mediated initiation. Safety concerns related to the thermal instability and potential explosiveness of peroxides remain a primary drawback. Additionally, the harsh reaction conditions often required for peroxide decomposition can severely compromise functional group tolerance and selectivity, thereby limiting their applicability toward structurally complex substrates.

### Alkyl-Substituted Peroxides

Alkyl-substituted peroxides can serve as efficient alkyl radical precursors for the C3-alkylation of coumarins. In the presence of metal catalysts such as copper or iron, these peroxides decompose to generate alkyl radicals. The resulting radical species adds regioselectively to the C3 position of the coumarin scaffold, affording the typical benzylic radical intermediate. Subsequent metal-mediated oxidation followed by rearomatization furnishes the desired product, with regeneration of the catalyst. Using this approach, Zou and co-workers reported the synthesis of C3-methylated coumarins employing di-*tert*-butyl peroxide (DTBP) as the radical source and CuCl (10 mol %) as the catalyst in chlorobenzene at 140 °C for 10 h (Scheme 11).^115^ The protocol exhibited good functional group tolerance, accommodating both electron-donating and electron-withdrawing substituents to afford the desired products in moderate yields (50–68%). Notably, C4-substituted coumarins bearing methyl or methoxy groups also proved compatible, providing the corresponding products in 60–68% yield. The proposed mechanism involves metal-promoted homolysis of DTBP to generate *tert*-butoxy radicals, which subsequently undergo β-scission to produce acetone and the corresponding methyl radical species. Control experiments demonstrated the crucial role of the metal catalyst, as only 18% of the product was formed in its absence. Furthermore, the addition of TEMPO completely suppressed the reaction and the detection of the TEMPO-CH_3_ adduct by LC–MS provides strong evidence for the involvement of methyl radicals.

**Scheme 11:**
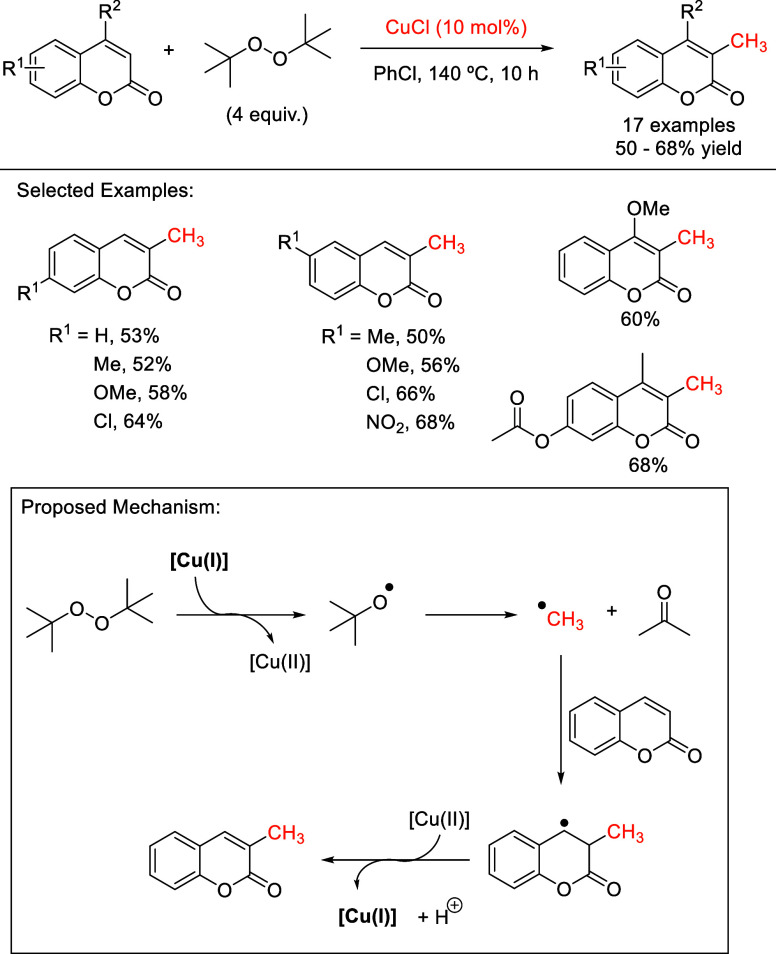
Radical C3-Methylation of Coumarins Using Peroxides

In 2019, Jin and co-workers significantly expanded the scope of this strategy using an iron catalyst in combination with alkyl diacyl peroxides as radical precursors (Scheme 12).^116^ Optimal conditions were identified using Fe­(OTf)_3_ (5 mol %) in dioxane at 70 °C under an inert nitrogen atmosphere, notably without the need for external ligands or additives. Control experiments confirmed that both the iron catalyst and an oxygen-free environment are crucial for achieving efficient reactivity. Under these conditions, primary alkyl diacyl peroxides proved to be the most effective substrates, affording the corresponding C3-alkylated coumarins in good yields (58–80%). Secondary peroxides were also compatible, although with slightly lower efficiency (50–58%). In contrast, tertiary alkyl diacyl peroxides failed to deliver the desired products, likely due to their reduced stability and propensity for competing decomposition pathways. The methodology tolerated peroxides bearing functional groups such as aryl (65%) and alkenyl (69%), highlighting its versatility. The scope with respect to the coumarin substrates was also systematically investigated. A wide variety of substituted coumarins underwent C3-alkylation, providing the desired products in moderate to good yields (40–83%). Both electron-donating and electron-withdrawing substituents were well tolerated, including methyl (81%), methoxy (47–78%), halogens (68–71%), and nitro (51%) groups. Notably, substrates containing free hydroxyl functionalities also participated effectively (40%). Additionally, coumarins bearing a C4-methyl substituent were compatible, affording products in moderate yields (48–63%). The mechanism for this transformation is proposed to proceed via an iron-mediated single-electron transfer (SET) coupled with the decarboxylation of the diacyl peroxide to generate the alkyl radical. The involvement of radical intermediates is strongly supported by trapping experiments, including the detection of TEMPO-radical adducts by ESI-MS. The resulting alkyl radical subsequently undergoes addition to the C3 position of the coumarin framework, followed by oxidation and deprotonation to furnish the final alkylated product while regenerating the active iron catalyst.

**Scheme 12:**
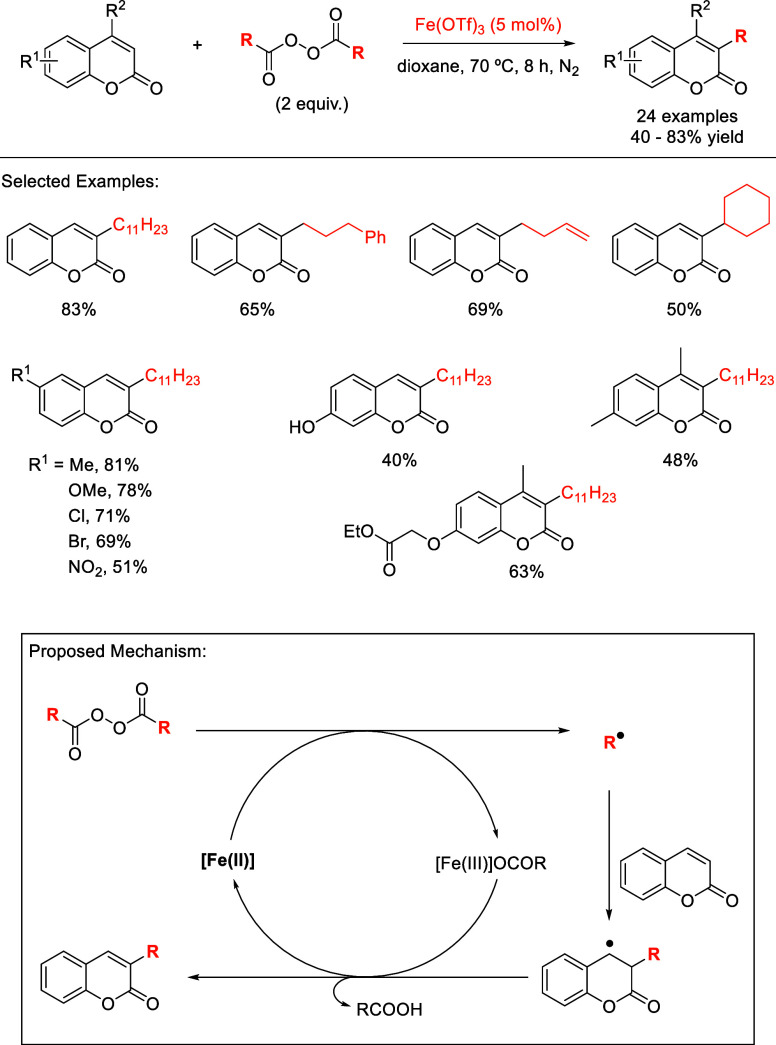
Radical C3-Alkylation of Coumarins Using Diacyl Peroxides

### Miscellaneous

In 2015, Miranda and co-workers investigated the feasibility of direct C3-alkylation of coumarins via a xanthate-based radical approach (Scheme 13).^117^ This strategy relies on the generation of α-carbonyl alkyl radicals from xanthates using dilauroyl peroxide (DLP) as the radical initiator. Under the optimized conditions, coumarin substrates were treated with xanthate (2 equiv) in dichloroethane (1,2-DCE) under microwave irradiation, with the portionwise addition of DLP (1.5 equiv), affording the desired products in generally moderate yields (12–77%). A variety of coumarins and xanthate was examined to assess the scope of the methodology. The reaction tolerates various substituents at the C7 position, including methoxy (12–77%), methyl (58%), and free hydroxy groups (33–70%). Structurally diverse xanthates were also compatible with the reaction conditions, including those derived from ethyl ester (33–58%), morpholine (48–77%), piperidine (37–63%), and more complex functionalities such as malonates (12–19%) and hydantoins (30–70%). The mechanism is proposed to proceed through thermal decomposition of DLP, generating carbon-centered radicals from the xanthate precursor. The resulting alkyl radical selectively adds to the C3 position of the coumarin, forming a stabilized benzylic radical intermediate. In the presence of excess peroxide, this intermediate undergoes oxidation to the corresponding carbocation, followed by deprotonation to furnish the final C3-alkylated product. This method was effective in incorporating more complex carbonyl structures into coumarins than previously reported works. However, these advantages are offset by a strict substrate-dependent scope limitation, poor atom economy and the safety hazards innate to peroxide-mediated transformations.

**Scheme 13:**
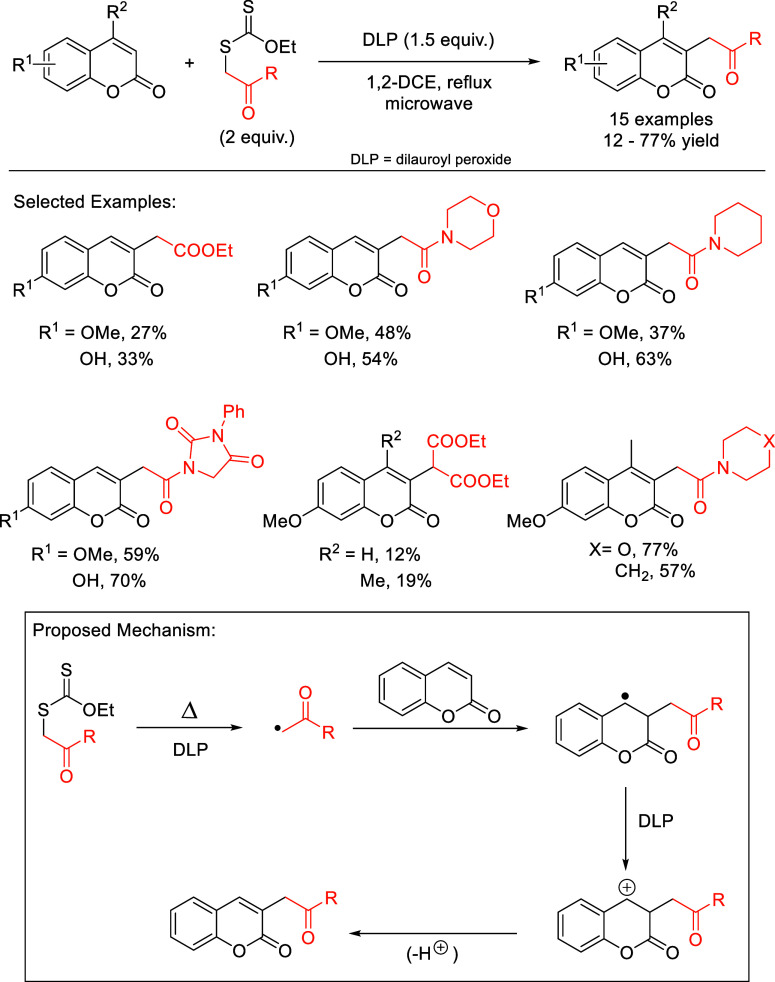
Radical C3-Alkylation of Coumarins Using Xanthate

An efficient protocol for radical alkylation of activated alkenes employing readily available and bench-stable organoboronic acids as alkyl radical precursors was reported by Liu and co-workers. Reaction optimization was conducted using *N*-arylacrylamides as model substrates (Scheme 14).^118^ The optimal conditions involve the use of a large excess of alkylboronic acid (5 equiv) in a mixed acetic acid/acetonitrile (AcOH/MeCN) solvent system, with trifluoroacetic acid (TFA) as an additive, under an oxygen atmosphere (1 atm) at 110 °C. Control experiments established that both molecular oxygen and the acidic additive are crucial for the transformation, as reactions carried out under a nitrogen atmosphere or in the absence of acid resulted in significantly diminished yields or no conversion. With the optimized conditions in hand, the scope of the methodology was extensively explored and successfully extended to a variety of activated alkenes, including coumarins. In particular, the regioselective C3-alkylation of coumarins was achieved in good yields (62–70%). Notably, C4-methylated coumarins bearing a free hydroxyl group at the C7 position provided the desired product in 70% yield. The authors did not evaluate the scope with respect to the boronic acid substrates, limiting the applicability of the method only to a cyclohexane derivative. Mechanistic investigations support a radical pathway. The addition of the radical scavenger TEMPO completely suppresses product formation and results in the isolation of the corresponding TEMPO-radical adduct, providing evidence for the involvement of alkyl radical intermediates. Based on these observations, a plausible mechanism initiates by the reaction of the alkylboronic acid with molecular oxygen, generating an alkyl radical along with a peroxyboron species. The alkyl radical adds to the C3 position of the coumarin, forming a benzylic radical intermediate. A subsequent hydrogen atom transfer step furnishes the final product and completes the radical chain process. This method stands out as a metal-free strategy employing O_2_ as the terminal oxidant, thereby avoiding the need for conventional stoichiometric oxidants or transition-metal catalysts.

**Scheme 14:**
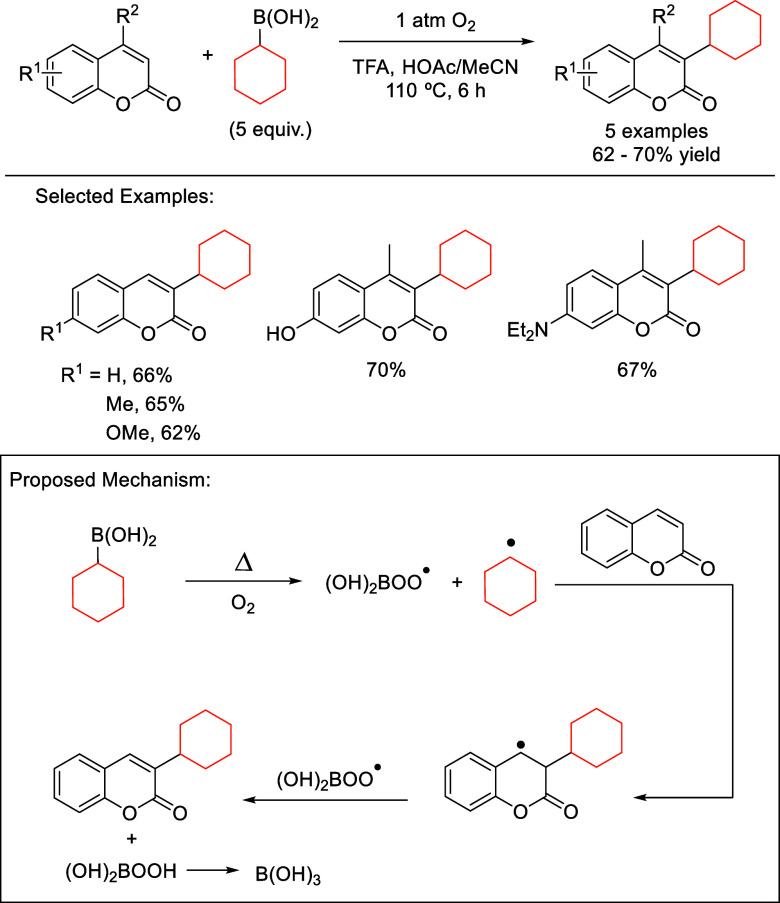
Alkylation of Coumarin with Organoboronic Acid

Carboxylic acids were employed by the Jafarpour group as alkyl radical precursors in a direct decarboxylative cross-coupling of coumarins under metal-free conditions (Scheme 15).^119^ The transformation proceeds under oxidative conditions using potassium persulfate (K_2_S_2_O_8_) as the oxidant and potassium carbonate (K_2_CO_3_) as the base. Optimal results were obtained with coumarin and pivalic acid (2 equiv) as model substrates in a H_2_O/MeCN (5:1) solvent system, affording the desired product in 74% yield. The scope of the reaction was comprehensively investigated. A broad range of aliphatic carboxylic acids, including primary, secondary, and tertiary substrates, were successfully employed, delivering the corresponding C3-alkylated coumarins in moderate to good yields (47–74%). Notably, challenging substrates such as cyclopropyl carboxylic acids performed well without undergoing ring opening, providing the desired product in 67% yield. In addition, simple acids such as acetic acid enabled methylation at the C3 position in 47% yield. The methodology also exhibited good tolerance toward variously substituted coumarins. Electron-donating groups, such as methyl and methoxy substituents, were well tolerated, affording products in high yields (70–82%), while halogen substituents were also compatible, giving moderate to good yields (54–73%). Furthermore, 4-methyl-substituted coumarins were suitable substrates, providing the desired alkylated product in 55% yield. Electron-withdrawing substituents significantly affected the reaction outcome, as evidenced by the low yields obtained for a nitro-substituted coumarin (20%). Additionally, sterically hindered substrates, such as adamantyl derivatives, were not compatible under the optimized conditions. Mechanistic investigations suggest a radical pathway. Control experiments employing TEMPO as a radical scavenger completely suppressed product formation, providing strong evidence for the involvement of radical intermediates. The proposed mechanism begins with the thermal decomposition of persulfate, generating sulfate radical anions. These species abstract a hydrogen atom from the carboxylic acid to form a carboxyl radical, which rapidly undergoes decarboxylation to yield the corresponding alkyl radical. The resulting alkyl radical subsequently adds to the C3 position of the coumarin, forming a new carbon-centered intermediate. This intermediate is then oxidized, followed by deprotonation, to furnish the final C3 alkylated product. Overall, this methodology stands out by enabling the installation of simple alkyl groups from readily available and abundant carboxylic acids through a transition-metal-free protocol.

**Scheme 15:**
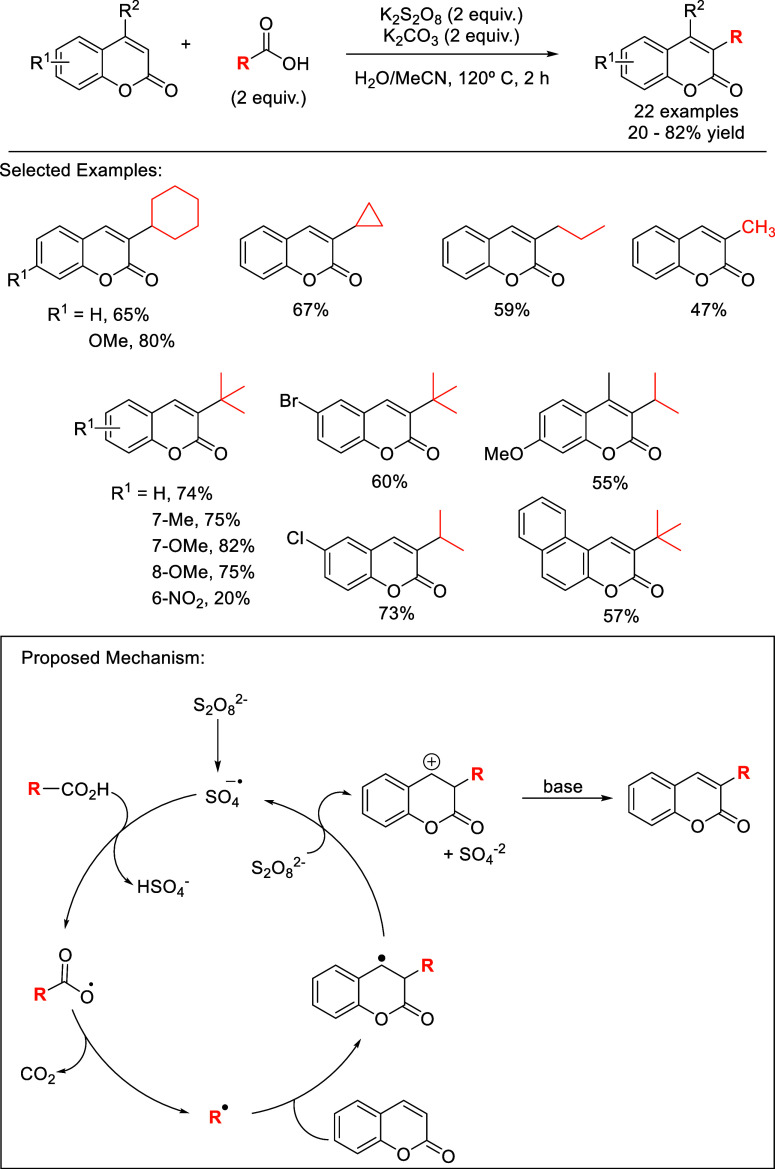
Alkylation of Coumarin with Carboxylic Acids

In 2019, Duan and co-workers reported an iron­(II)-catalyzed protocol for the direct C–H cyanoalkylation of coumarins using cyclobutanone oxime esters as radical precursors. This methodology offers a regioselective and efficient approach for accessing C3-cyanoalkylated derivatives under redox-neutral conditions (Scheme 16).^120^ Optimization studies identified iron phthalocyanine (15 mol %) as the catalyst, with acetonitrile as the solvent at 100 °C under a nitrogen atmosphere. Under these conditions, using cyclobutanone *O*-benzoyl oxime ester and coumarin (1.5 equiv), the desired cyanoalkylated product was obtained in 72% yield. Other iron salts, including Fe­(OAc)_2_, FeCl_2_, and FeCl_3_, were also evaluated but proved less effective. The substrate scope with respect to coumarins was systematically investigated, demonstrating good functional group tolerance. A variety of substituted coumarins bearing both electron-donating and electron-withdrawing groups at different positions were well tolerated, affording the corresponding products in moderate to good yields (30–72%). A C4-methyl-substituted coumarin delivered the desired alkylated product in 42% yield. Notably, functional groups such as alkenes, free hydroxyl groups, and fused heterocycles were compatible with the reaction conditions, affording the respective products in 48%, 35%, and 30% yields. Regarding the radical precursors, a broad range of cyclobutanone oxime esters bearing different substituents was successfully employed. Aryl-substituted oxime esters containing methyl, chloro, bromo, and ester functionalities efficiently generated cyanoalkyl radicals and furnished the desired products in 45–79% yield. Alkyl-substituted oxime esters were also suitable substrates, providing the corresponding products in 48–62% yield, thereby further expanding the scope of the transformation. The involvement of a radical pathway was supported by control experiments. The addition of TEMPO as a radical scavenger completely suppressed product formation and led to the isolation of a cyanoalkyl-TEMPO adduct. Based on these findings, a plausible mechanism involves single-electron reduction of the oxime ester by Fe­(II), generating an iminyl radical that undergoes C–C bond cleavage to form a cyanoalkyl radical. This intermediate selectively adds to the C3 position of the coumarin core, followed by oxidation and deprotonation to furnish the final product.

**Scheme 16:**
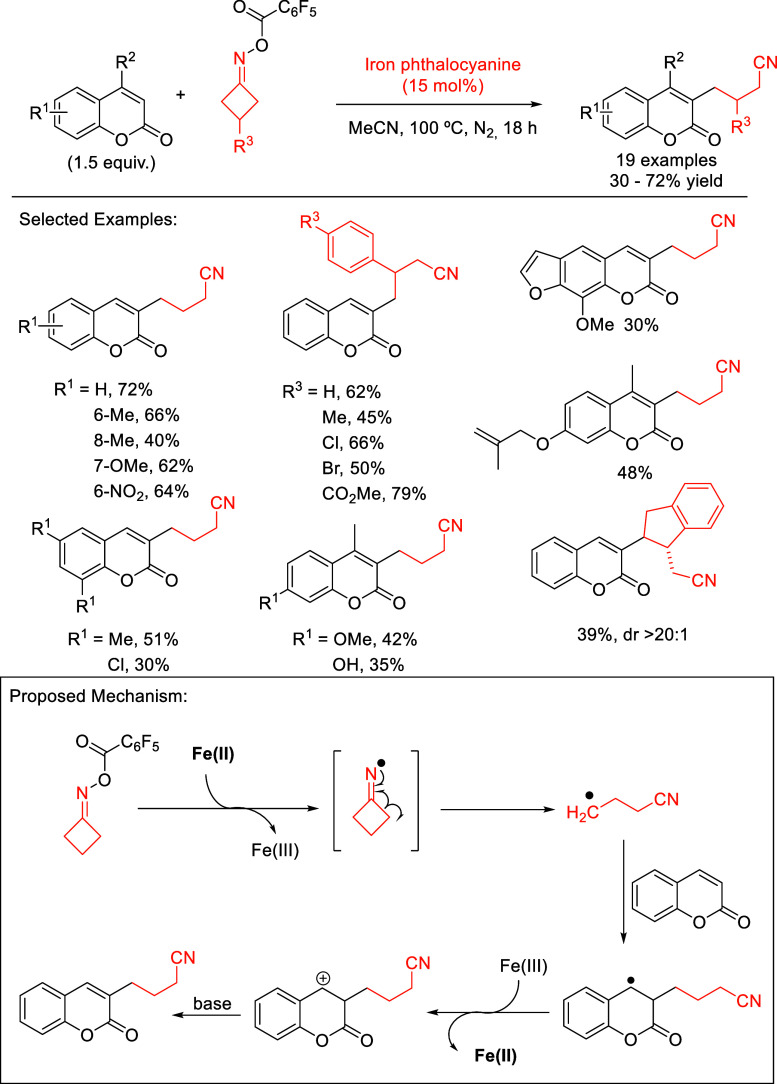
Alkylation of Coumarin with Oxime Esters

As presented throughout this section, thermally driven protocols have significantly contributed to the development of C3-alkylation reactions of coumarins. Nevertheless, these approaches generally rely on elevated temperatures, a large excess of coupling partners and stoichiometric oxidants, which may compromise chemical efficiency and substrate compatibility. Consequently, the development of radical-based methodologies operating under mild conditions remains highly desirable, as such strategies could improve reaction efficiency while expanding functional group tolerance.

## Photoredox Reactions Conditions

The advent of visible-light-driven transformations has ushered in a new era in radical chemistry, establishing photoredox strategies as a powerful platform for the generation of carbon-centered radicals under mild conditions.^8–10^ These methods generally circumvent the need for elevated temperatures and stoichiometric oxidants, thereby enhancing functional group tolerance and improving the overall sustainability of the processes. As a result, photoredox transformations have substantially expanded the range of viable radical precursors and enabled access to a diverse array of carbon-centered species, including the particularly challenging unstabilized alkyl radicals.^4,121–125^ Functionalization of coumarins has increasingly benefited from the unique features of visible-light photoredox strategies. In particular, C3-regioselective radical alkylation reactions have been developed using a set of radical precursors, including alkyl iodides, activated alkyl bromides, methylamines, Katritzky salts, and redox-active esters. Although the scope of these transformations remains limited in terms of the variety of radical precursors employed, the reported methodologies highlight the significant potential of photoredox strategies to promote mild, selective, and efficient C–C bond formation in coumarin frameworks.

### Redox-Active Esters

Carboxylic acids are attractive radical precursors in organic synthesis, particularly due to their abundance and ready availability compared with alkyl halides.^126–129^ However, their widespread application in radical chemistry remains limited by intrinsic challenges related to reactivity and selectivity. These drawbacks primarily originate from the high strength of the C–O bond and the relatively high redox potentials associated with carboxylic acids. A more general strategy involves the generation of alkyl radicals from carboxylic-acid-derived redox-active esters, such as *N*-(acyloxy)­phthalimides (NHPI esters), through single-electron-transfer-induced decarboxylative processes.^130–134^ These NHPI esters can be readily prepared from the corresponding carboxylic acids and *N*-hydroxyphthalimide using standard protocols (Scheme 17). Moreover, the phthalimide byproduct generated from NHPI esters can be recovered and recycled for the synthesis of new redox-active esters, enhancing the sustainability of these methodologies.^135–137^

**Scheme 17:**
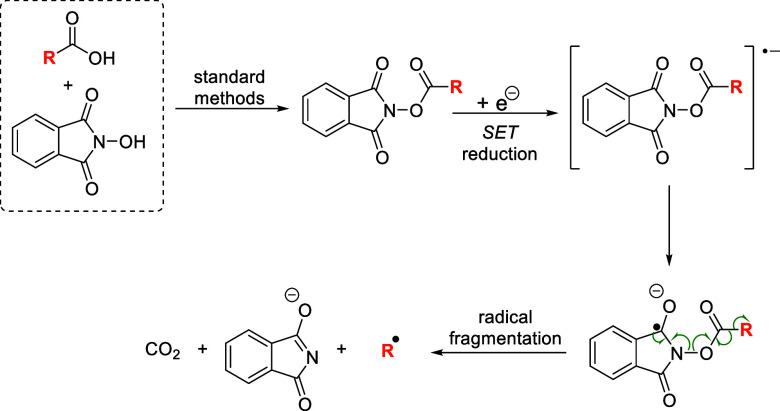
Single-Electron-Transfer-Induced Decarboxylative Radical Generation from NHPI

In 2019, Jin and co-workers explored the potential of *N*-hydroxyphthalimide (NHPI) esters as alkyl radical precursors in a visible-light-mediated decarboxylative process to achieve C3-alkylation of coumarins (Scheme 18).^138^ The reaction conditions for this transformation are based on Ir­(ppy)_3_ (2 mol %) as a photocatalyst under white LED irradiation and acidic conditions. A wide range of primary, secondary, and tertiary NHPI esters were well tolerated, including methyl, heterocyclic, and natural amino acid derivatives (47–92%). This method proved to be particularly efficient for coumarins bearing electron-donating groups, such as methyl (75%) and methoxy (85%) substituents at the 6-position of the aromatic ring. As expected, the presence of electron-withdrawing substituents in the coumarin substrate, such as halogens, disfavored the transformation, resulting in moderate yields (42–51%). Following this trend, disubstituted coumarins containing two electron-withdrawing groups underwent the decarboxylative coupling process to afford the desired products in relatively low yields (30–36%). These observations are consistent with the proposed mechanism, which involves the formation of benzylic radicals and carbocations as key intermediates. The radical nature of the mechanism was supported by control experiments using TEMPO and BHT as radical scavengers, both of which completely suppressed the reaction. In addition, Stern–Volmer studies indicated that the excited state [Ir­(ppy)_3_]* could be quenched by alkyl NHPI esters, suggesting that the reaction proceeds through an oxidative quenching pathway. Although this photoredox strategy enables selective and efficient alkylation of coumarins under mild conditions, the use of an expensive iridium complex and two equivalents of the NHPI ester represents notable drawbacks.

**Scheme 18:**
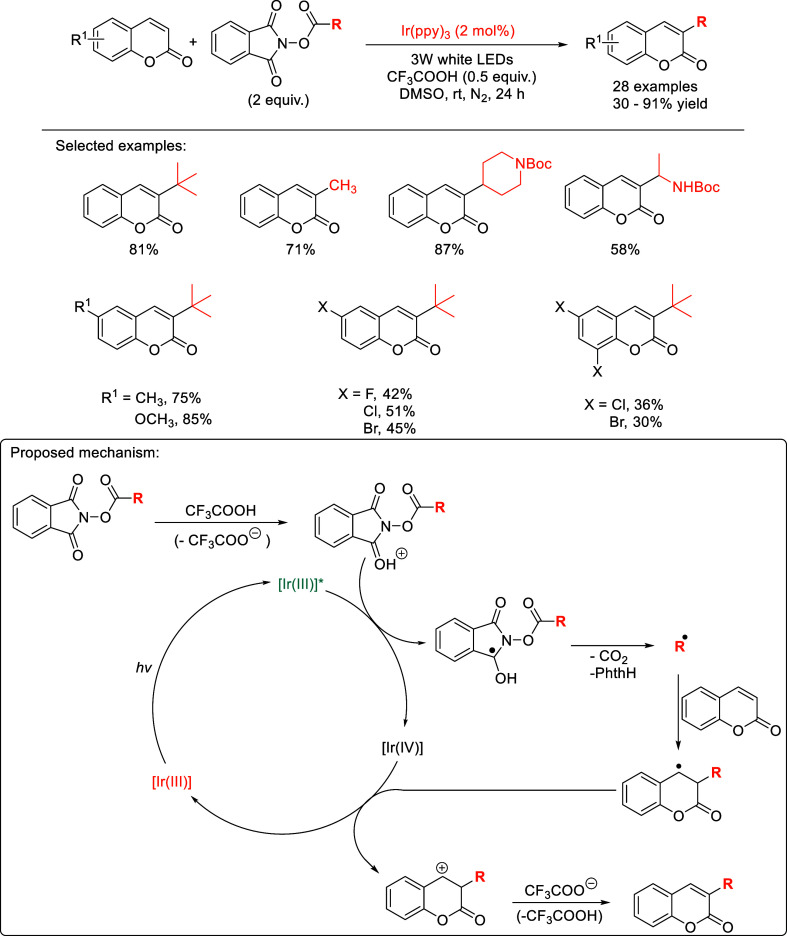
Photoredox-Mediated C3-Radical Alkylation of Coumarins Using NHPI Esters

Concurrently, a similar photoredox strategy was developed by Dong and co-workers, offering the advantages of a shorter reaction time (2 h), a very low catalyst loading of Ru­(bpy)_3_Cl_2_ (0.2 mol %), and the use of only 1.3 equiv of the NHPI ester substrate (Scheme 19).^139^ The reaction was conducted under basic conditions and sunlight irradiation, although the use of blue LEDs provided comparable yields. This methodology tolerated a broad substrate scope of NHPI alkyl esters, affording desired products in 30–91% yield, including a pharmaceutical gemfibrozil-derived NHPI ester. Notably, coumarins bearing a methyl substituent at the C4 position (*R* ^2^ = Me), afforded the desired product in good yields (*R* ^1^ = NEt_2_, 54%). In addition, a one-pot synthetic strategy proved effective, involving the in situ formation of NHPI alkyl esters from free carboxylic acids followed by the radical coupling protocol. The method was also efficiently applied on a 10 mmol scale, although the product yields were slightly lower than those obtained using the original protocol.

**Scheme 19:**
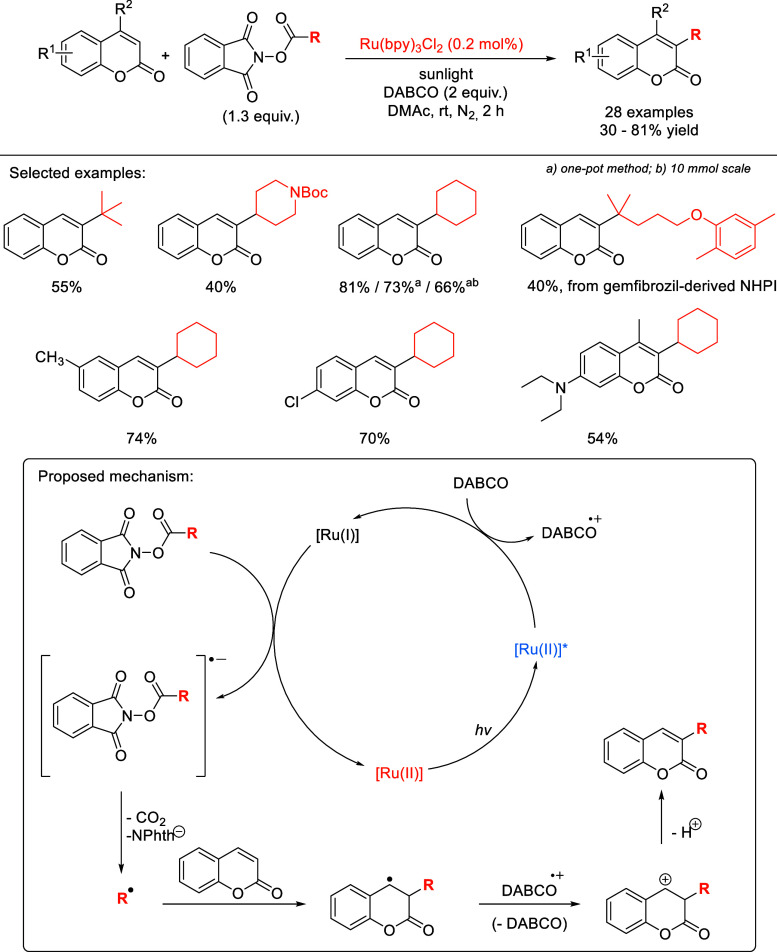
Ruthenium-Photocatalyzed C3-Alkylation of Coumarins Using NHPI Esters

In 2023, Li and co-workers reported a one-pot Wittig/cyclic condensation/radical alkylation for the synthesis of 3-alkyl coumarins from salicylaldehydes (Scheme 20).^140^ The reaction sequence begins with a Wittig reaction between salicylaldehydes and ethyl (triphenylphosphoranylidene)­acetate, followed by photochemical isomerization and intramolecular cyclization to generate coumarin intermediates. In the second step, performed without purification of the intermediate, the resulting coumarin undergo visible-light-photocatalyzed alkylation using *fac*-Ir­(ppy)_3_ (2 mol %) as the photocatalyst, DABCO (2 equiv) as an additive, and a large excess of *N*-hydroxyphthalimide esters (3 equiv) as radical precursors. Tertiary, secondary, and primary alkyl NHPI esters were compatible with this protocol, however, tertiary alkyl radical sources generally afforded higher yields (up to 74% over two steps), likely due to their enhanced radical stability. Isobutyl-, cyclohexyl-, and *n*-heptanyl-substituted substrates were converted into the corresponding 3-alkyl coumarins in moderate yields (45–65% over two steps). Control experiments confirmed the essential role of visible light in the ring-closure process of the first step, which did not proceed in the absence of visible-light irradiation, supporting a mechanism involving Wittig olefination, photoinduced isomerization, cyclization, and subsequent radical alkylation.

**Scheme 20:**
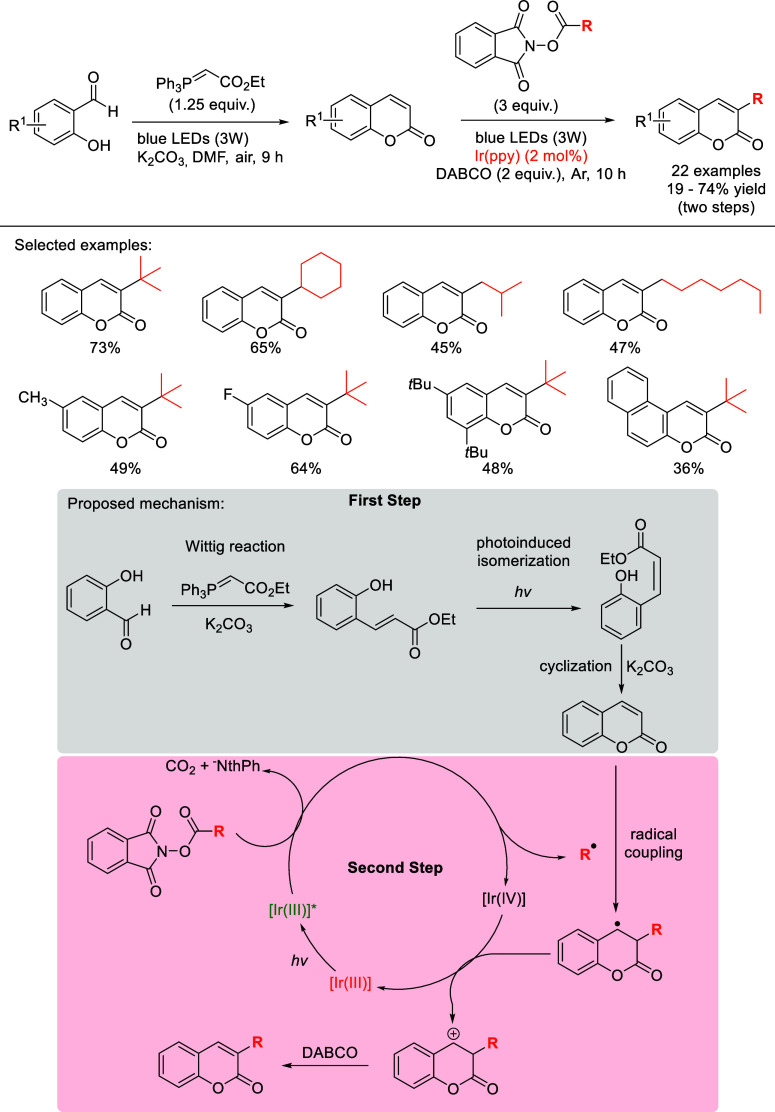
One-Pot Wittig/Cyclic Condensation/Radical Alkylation

Earth-abundant transition-metal complexes, such as copper, iron, and cobalt, offer a sustainable alternative to iridium and ruthenium photocatalysts under visible-light conditions. Their application in organic synthesis, however, remains limited due to challenges in tuning redox properties and excited-state lifetimes for efficient photoredox transformations. Wang and co-workers reported a copper-photocatalyzed decarboxylative coupling of alkyl NHPI esters, focusing on the alkylation of *N*-heteroarene substrates. Notably, the methodology was extended to a single example of coumarin alkylation, which was obtained in 45% yield (Scheme 21).^141^

**Scheme 21:**
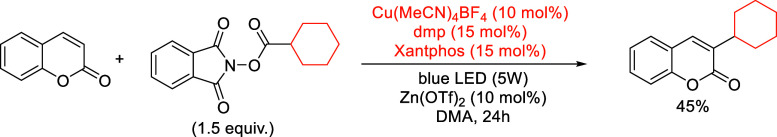
Copper-Photocatalyzed C3-Alkylation of Coumarins Using NHPI Esters

A transition-metal and oxidant-free photocatalytic strategy for the regioselective C3 alkylation of coumarins was developed by Dong’s group.^142^ The reaction is mediated by triphenylphosphine (PPh_3_) and sodium iodide (NaI) under blue LED irradiation, using alkyl *N*-hydroxyphthalimide (NHPI) esters as alkylating agents (Scheme 22). Under the optimized conditions, a broad range of NHPI esters derived from primary, secondary, and tertiary carboxylic acids were well tolerated, affording 3-alkylated coumarins in moderate to high yields (36–90%). The protocol proved effective for substituted coumarins, showing good functional group tolerance toward methoxy (86%), methyl (90%), chloro (69%), and fused ring (46%) substituents. Furthermore, coumarins bearing a methyl substituent at the C4 positions (*R* ^2^ = Me) delivered the desired products in good yields (*R* ^1^ = OMe, 80%; *R* ^1^ = NEt_2_, 48%). Notably, the reaction could be successfully scaled up using the model substrate, although with a slightly decreased isolated yield (72%). The utility of this method was also extended to quinoxalinones and heteroaryl-fused *N*-heterocycles, highlighting its wide substrate scope. Control experiments revealed that the addition of TMEDA to the PPh_3_/NaI system induced a redshift in the absorption profile, likely due to TMEDA acting as a ligand that significantly enhances the photoactivity of the system. Based on experimental observations and literature precedents, the authors proposed a mechanism in which a photoactive chromophore, formed in situ from PPh_3_, NaI, and the NHPI ester, undergoes single-electron transfer (SET) under blue light irradiation. Electron transfer from the iodide anion to the alkyl NHPI ester generates a phosphorus-stabilized iodine radical species along with an alkyl radical via decarboxylation, accompanied by the formation of sodium phthalimide. The resulting alkyl radical selectively adds to the C3 position of the coumarin, followed by subsequent oxidation and deprotonation to furnish the final product. Overall, this strategy enables highly regioselective alkylation of coumarins under mild conditions, without the need for metals or external oxidants.

**Scheme 22:**
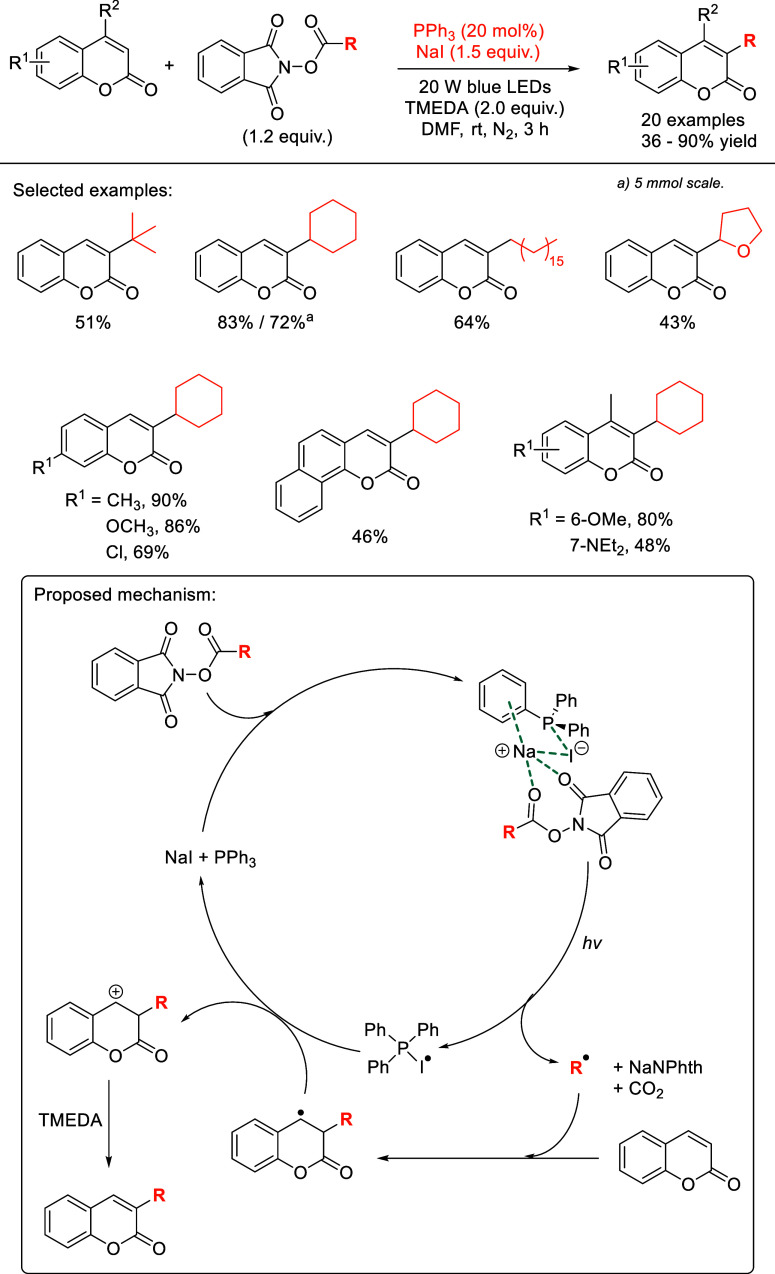
Transition-Metal-Free Photocatalytic Strategy Using NHPI Esters

In 2022, Tan and co-workers reported a transition-metal-free photoredox system based on an electron donor–acceptor (EDA) complex for the generation of alkyl radicals from *N*-hydroxyphthalimide (NHPI) esters.^143^ Under blue or violet LED irradiation, coumarins reacted smoothly with a broad range of NHPI esters in the presence of DIPEA (0.2 equiv) and an appropriate alkali metal carbonate (1.2 equiv) to afford the desired alkylated product (Scheme 23). Optimization studies revealed that Li_2_CO_3_ generally performed best for primary and secondary alkyl radical precursors, whereas Cs_2_CO_3_ was superior for tertiary radicals. The method exhibited good functional-group tolerance, working with primary, secondary, tertiary, heterocyclic, halogenated, and α-amino acid based NHPI esters to afford alkylated coumarins in moderate to excellent yields (20–91%). Notably, a gram-scale reaction using a gemfibrozil-derived NHPI ester delivered the target product in 88% yield, highlighting the robustness and scalability of the protocol. In contrast to conventional EDA-based transformations, which typically require stoichiometric amounts of donors or acceptors, this work represents a significant advance by establishing a donor-catalyzed EDA system in which DIPEA (20 mol %) operates catalytically as the electron donor. Mechanistic studies strongly supported an EDA-mediated photoredox pathway. The EDA complex consists of DIPEA as the donor catalyst, the NHPI ester as the acceptor, and an alkali metal carbonate acting concurrently as a stabilizer and an electron-transfer mediator. Density functional theory (DFT) calculations indicated that the direct formation of the ground-state DIPEA-NHPI ester complex is thermodynamically unfavorable. However, in the presence of a metal carbonate, the formation of a three-component complex becomes energetically favorable. The associated decrease in free energy corroborates the stabilizing role of metal carbonates through electrostatic interactions. Beyond coumarin functionalization, the authors demonstrated the general applicability of this DIPEA-based EDA catalysis system across several net redox-neutral radical transformations, including alkylation–cyclization cascades of 2-isocyanobiphenyls to form phenanthridines and radical addition–cyclization cascades of *N*-arylacrylamides to afford quaternary oxindoles.

**Scheme 23:**
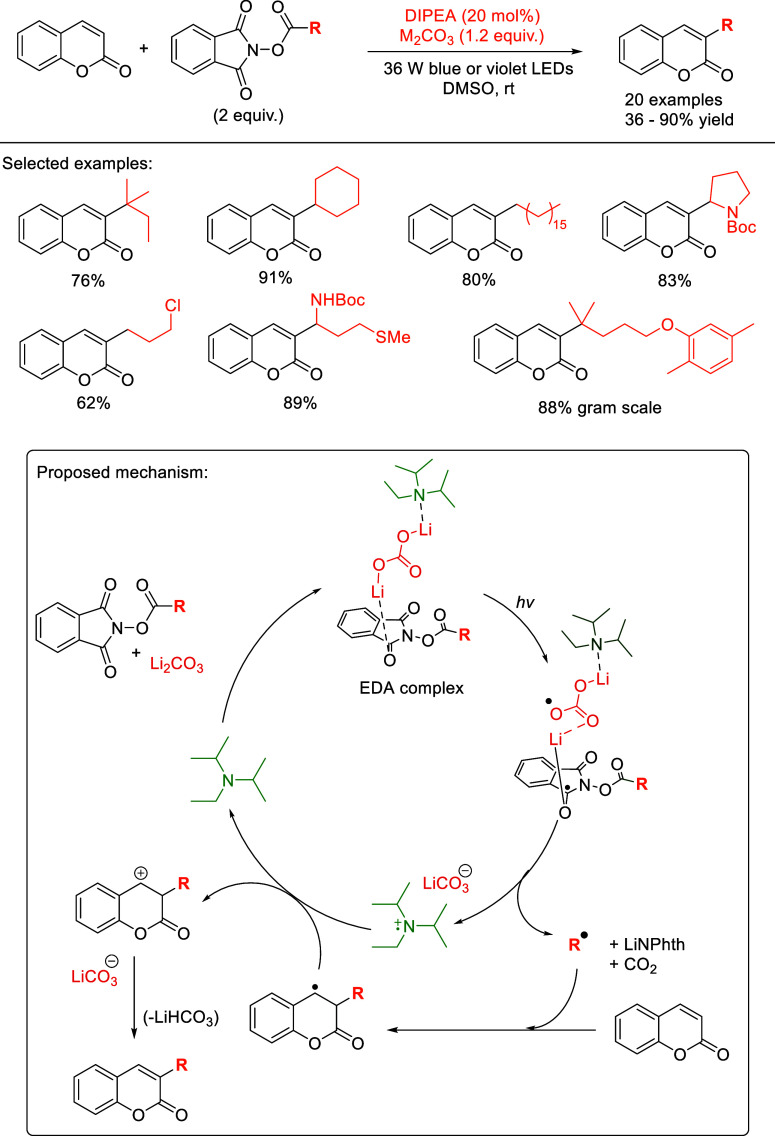
EDA Complex Strategy for the Alkylation of Coumarins Using NHPI Esters

Subsequently, He and co-workers employed Na_2_S as a catalytic electron donor to form an electron donor–acceptor (EDA) complex with NHPI esters generated in situ from carboxylic acids (Scheme 24).^144^ Upon visible-light irradiation (455 nm LEDs), this EDA complex undergoes single-electron transfer, enabling efficient alkyl radical generation without the need for an external photocatalyst or stoichiometric additives, thereby overcoming several limitations associated with conventional photocatalytic decarboxylative alkylation reactions. The substrate scope demonstrated broad functional-group tolerance. A wide range of primary, secondary, and tertiary alkyl carboxylic acids were successfully employed (58–96%), including linear, cyclic, and sterically hindered substrates such as adamantyl (96%) and gemfibrozil (86%) derivatives. Coumarins bearing either electron-donating or electron-withdrawing substituents at various positions of the aromatic ring were also compatible, affording the desired products in good to excellent yields (74–92%). In addition, the reaction was performed on gram-scale using the model substrate without a significant loss of efficiency. Mechanistic investigations supported a radical pathway. The reaction was completely suppressed in the presence of radical scavengers such as TEMPO and BHT. UV–vis spectroscopy revealed characteristic absorption changes consistent with the formation of an EDA complex between the NHPI ester and Na_2_S. Light on/off experiments and a low quantum yield (2.7%) ruled out a radical chain mechanism, confirming that continuous photoexcitation is required. Based on these results, the authors proposed a mechanism in which visible-light excitation of the EDA complex induces single-electron transfer to generate an alkyl radical and a sulfur-centered radical species. The alkyl radical then adds regioselectively to the C3 position of the coumarin, forming a radical intermediate that is subsequently oxidized and deprotonated to furnish the final product, while simultaneously regenerating the sulfide catalyst.

**Scheme 24:**
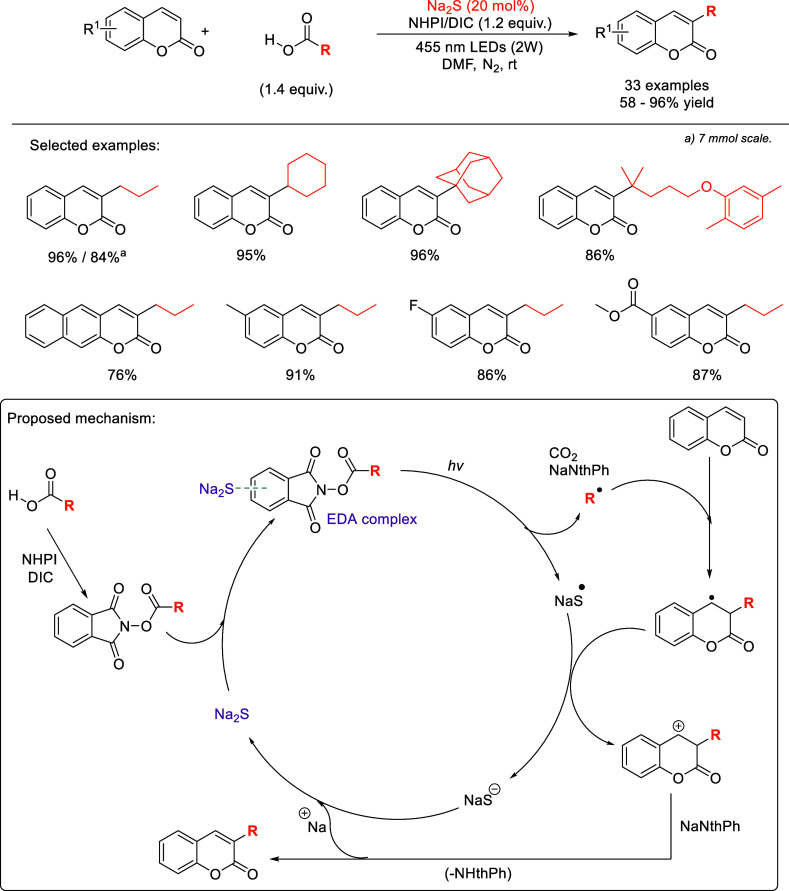
Regioselective Alkylation of Coumarins by Na_2_S-NHPI Esters-Based EDA Complex

Another notable example was reported by Yang and co-workers, who employed an olefin-linked two-dimensional covalent organic framework (2D-COF) as a heterogeneous photocatalyst to promote the efficient decarboxylative alkylation of heterocycles using *N*-hydroxyphthalimide (NHPI) esters under visible-light irradiation.^145^ The reaction proceeds under heterogeneous and acidic conditions, allowing facile catalyst recovery and avoiding the use of precious metal complexes or organic dyes, in contrast to traditional homogeneous photocatalytic systems. A wide variety of *N*-heteroaromatic substrates were successfully converted into the corresponding alkylated products in good to excellent yields. Remarkably, the synthetic utility of this methodology was also extended to a coumarin scaffold, delivering the desired product in 61% yield (Scheme 25). Mechanistic investigations supported a radical pathway, as evidenced by reaction inhibition in the presence of the radical scavenger TEMPO and further corroborated by electron paramagnetic resonance (EPR) studies.

**Scheme 25:**
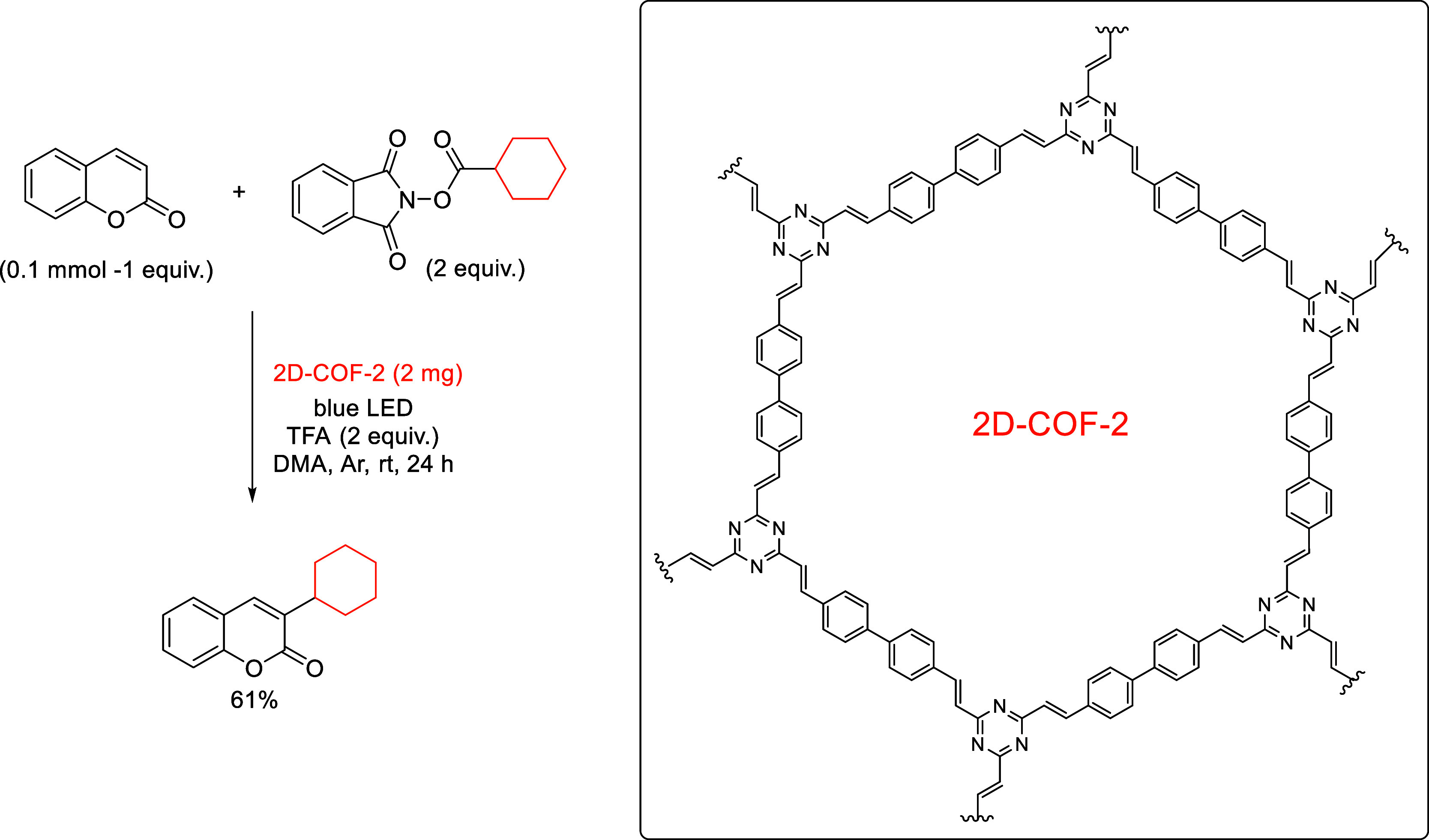
Covalent Organic Framework (2D-COF) as a Heterogeneous Photocatalyst

Overall, *N*-hydroxyphthalimide (NHPI) esters represent powerful and versatile platforms for the C3-alkylation of coumarins via photoredox strategies. As radical precursors, they exhibit good reactivity and efficiency in generating primary, secondary, and tertiary alkyl radicals under mild reaction conditions, including transition-metal-free protocols. Moreover, these reagents enable broad substrate scope studies, accommodating complex molecular architectures, such as natural product and drug derivatives. Nevertheless, a notable intrinsic drawback of this strategy is the need for additional synthetic steps to prepare the NHPI esters from the corresponding carboxylic acids. Furthermore, the recovery and recycling of the phthalimide byproduct generated during the decarboxylative coupling are crucial for enhancing the economic sustainability of these methodologies.

### Katritzky Salts

Katritzky salts, readily prepared from pyridium salts and primary amines, have emerged as efficient carbon-centered radical precursors in both traditional metal catalysis and photoredox chemistry.^146,147^ Their bench stability and favorable single-electron-transfer reactivity enable mild C–N bond cleavage, providing straightforward access to alkyl radicals from abundant amine feedstocks. In 2022, He and co-workers proposed that these pyridinium-activated primary amine salts could act as effective alkylating agents for coumarins under mild conditions.^148^ Reaction optimization identified [Ir­(dtbbpy)­(ppy)_2_]­PF_6_ (1 mol %) as the optimal photocatalyst under blue LED irradiation (Scheme 26). The presence of a base was crucial for driving the reactivity, with sodium acetate outperforming other inorganic and organic bases, while DMSO proved to be the most effective solvent. Control experiments confirmed that light, base, and photocatalyst were all required, supporting a photoredox catalytic pathway. A broad range of Katritzky salts derived from cyclic and acyclic aliphatic amines were well tolerated, affording the corresponding products in moderate to excellent yields (59–90%). In contrast, pyridinium salts derived from primary benzylic amines exhibited significantly lower reactivity (26–30%), and substrates derived from amino acids were ineffective. No other primary or tertiary substrates were evaluated. Regarding the coumarin scope, substrates bearing electron-donating substituents such as methoxy, ethoxy, and methyl on the aromatic ring were compatible with the reaction conditions, delivering good yields (72–80%). Coumarins bearing a methyl group at the C4 position (*R* ^2^ = Me) were also evaluated, providing the desired products in moderate yields (36–49%). To further demonstrate the synthetic utility of this methodology, gram-scale reactions were carried out without any loss of efficiency. The protocol proved highly effective for the late-stage functionalization of complex natural products and pharmaceuticals. Alkylation of furanocoumarins, including methoxsalen and angelicin, proceeded with good efficiency and high regioselectivity (49–65%). Furthermore, Katritzky salts derivatives of the drug benazepril were successfully coupled with a variety of coumarins in up to 81% yield, highlighting the potential of this strategy for applications in medicinal chemistry and drug diversification.

**Scheme 26:**
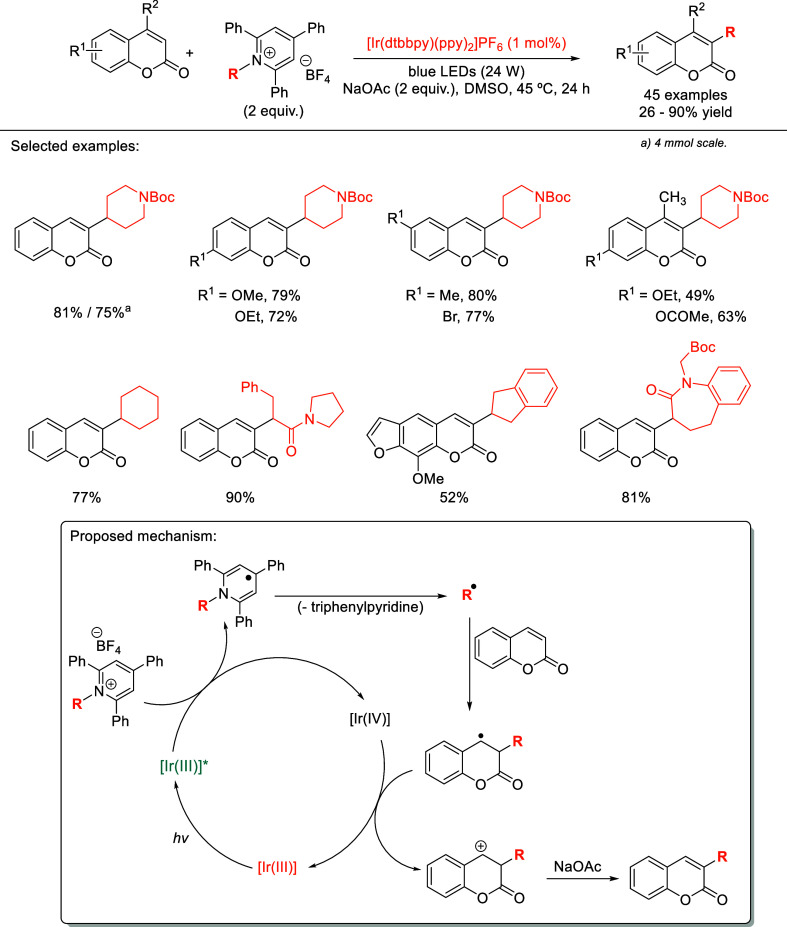
Katritzky Salts as Alkyl Radical Precursors for Alkylation of Coumarins

In general, these pyridinium salts are particularly valuable for converting amines into alkyl radical precursors, making them important tools in modern photoredox-mediated functionalization strategies. Their broad functional group tolerance further highlights their utility, enabling applications in the late-stage diversification of complex molecular architectures. Furthermore, some limitations remain, including the requirement for substrate prefunctionalization to install the pyridinium core and the unavoidable generation of stoichiometric pyridine-derived byproducts, which poses a challenge to the atom economy of the process.

### Methylamines

In 2023, Singh and co-workers evaluated a strategy for the generation of α-aminoalkyl radicals from tertiary *N,N*-dialkylanilines, enabling the direct aminoalkylation of coumarins.^149^ The optimized conditions employed Ru­(bpy)_3_Cl_2_·6H_2_O (2 mol %) as the photocatalyst, DBU (2 equiv) as the base, DMSO as solvent and molecular oxygen as the terminal oxidant under visible-light irradiation (Scheme 27). The *N,N*-dialkylaniline was used in a large excess of 4 equiv. Interestingly, replacing blue LEDs with white LEDs significantly improved the reaction efficiency, affording the model product in 84% yield. Control experiments established that light, the photocatalyst, and the base are all indispensable for product formation, underscoring the photoredox nature of the transformation. The substrate scope was investigated using a variety of *N,N*-dimethylaniline derivatives bearing both electron-donating and electron-withdrawing substituents. *para*-Alkyl-substituted anilines furnished the corresponding products in good to excellent yields (62–95%), whereas halogen-substituted analogues afforded moderate to good yields (36–75%). *meta*-Substituted anilines were also well tolerated, providing the aminoalkylated products in 61–67% yield, while *ortho*-substituted derivatives were unreactive, presumably due to steric hindrance. The scope of coumarins was likewise examined. Substitution at the 6-position of the coumarin core with methyl and methoxy groups resulted in high yields (75–82%), whereas halogen substituents led to reduced reactivity (22–48%), consistent with the increasing electronegativity of the substituents. A fused aromatic coumarin system was also compatible, affording the desired aminoalkylated product in 46% yield. In addition, a distinct mechanistic behavior was observed for 4-aminocoumarin substrates, whose enhanced stability inhibited their reactivity toward the α-aminoalkyl radical derived from *N,N*-dimethylaniline. However, in the absence of the base, an alternative iminium-ion-mediated pathway afforded the desired product in 55% yield.

**Scheme 27:**
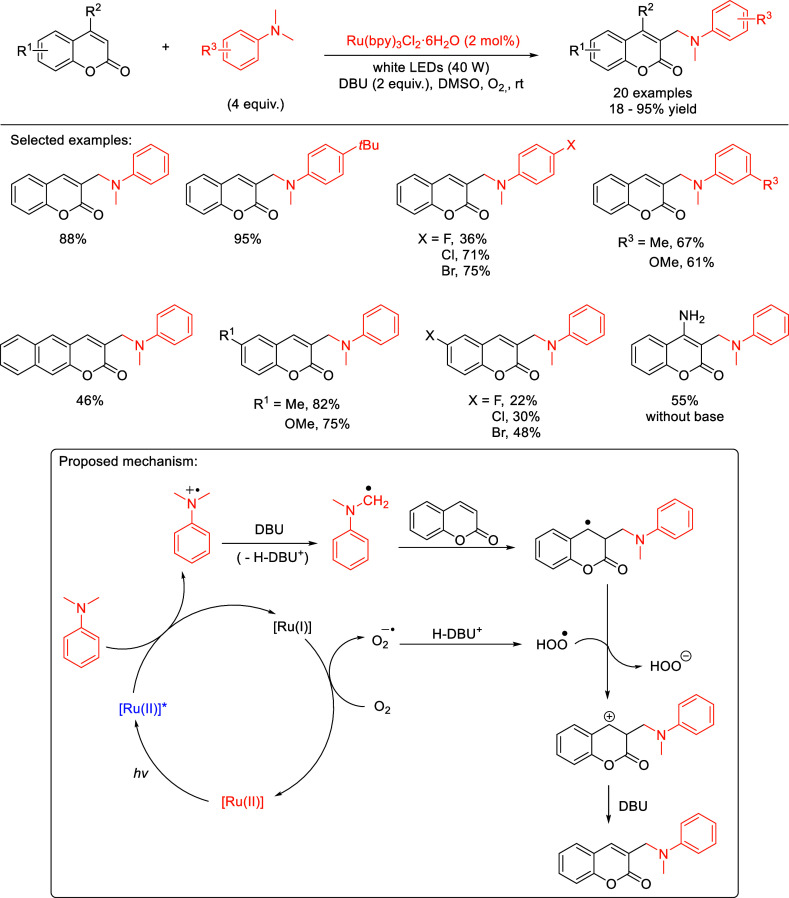
Ruthenium-Photocatalyzed Strategy to C3-Aminoalkylation of Coumarins

Mechanistic investigations, including radical-trapping experiments using TEMPO and BHT, strongly supported the involvement of radical intermediates. The proposed mechanism starts from photoexcitation of the Ru­(II) photocatalyst, followed by single-electron oxidation of *N,N*-dimethylaniline to generate a cation radical. Subsequent deprotonation affords the key α-aminoalkyl radical, which undergoes regioselective addition to the C3 position of the coumarin framework. Final oxidation and elimination steps deliver the α-aminoalkylated product, with molecular oxygen acting as the terminal oxidant. This approach overcomes key limitations of conventional methods for accessing C3-functionalized coumarins, which typically rely on prefunctionalized substrates or transition-metal-catalyzed dehydrogenative couplings employing hazardous peroxides and elevated temperatures.

A catalyst-free strategy for the direct C–H methylation of heteroarenes using readily available methylamines as the methyl radical source was reported by Sheng and co-workers.^150^ Methylation is a highly important transformation in medicinal chemistry due to the well-known “magic methyl effect”, whereby the introduction of a single methyl group can dramatically enhance biological activity, metabolic stability, and binding affinity of drug candidates.^151,152^ Despite its importance, direct C–H methylation of heteroaromatic compounds remains challenging and often relies on transition-metal catalysis, toxic methylating reagents, harsh reaction conditions, or expensive photocatalysts. In this work, the authors presented an alternative approach based on the photoactivation of an electron donor–acceptor (EDA) complex formed directly between the heteroarene substrate and a methylamine. Under visible-light irradiation, this EDA complex enables the generation of methyl radicals without the need for external photocatalysts, oxidants, or transition metals. The methodology was initially developed using 1-methylquinoxalin-2­(*1H*)-one as the model substrate and was subsequently extended to other classes of heteroarenes, including coumarins (Scheme 28). For the radical C3-methylation of coumarins, the optimal reaction conditions employed *N,N*-dimethylglycine (3 equiv) as the methyl source, a mixed DMA/H_2_O solvent system (1:1), K_3_PO_4_ (3 equiv) as the base, and irradiation at 410 nm (10 W) under a nitrogen atmosphere for 20 h. Notably, the presence of water proved crucial for enhancing the reaction efficiency. The substrate scope was further explored for coumarins bearing a variety of functional groups, including alkyl, alkoxy, fluoro, chloro, and ester substituents, as well as fused-ring systems, affording the corresponding C3-methylated products in moderate to good yields (39–77%). The protocol was also applicable to coumarins bearing a methyl substituent at the C4 position (*R* ^2^ = Me), delivering the desired product in 39% yield. Mechanistic investigations conducted with 1-methylquinoxalin-2­(*1H*)-one support a radical pathway initiated by photoexcitation of the EDA complex. UV–vis absorption studies revealed a redshift upon mixing the heteroarene and the methylamine, consistent with EDA complex formation. Furthermore, radical trapping experiments confirmed the involvement of radical intermediates. Based on these results, a plausible mechanism was proposed involving single-electron transfer within the EDA complex, generation of an α-amino radical, radical addition to the heteroarene, and subsequent protonation to furnish the methylated product.

**Scheme 28:**
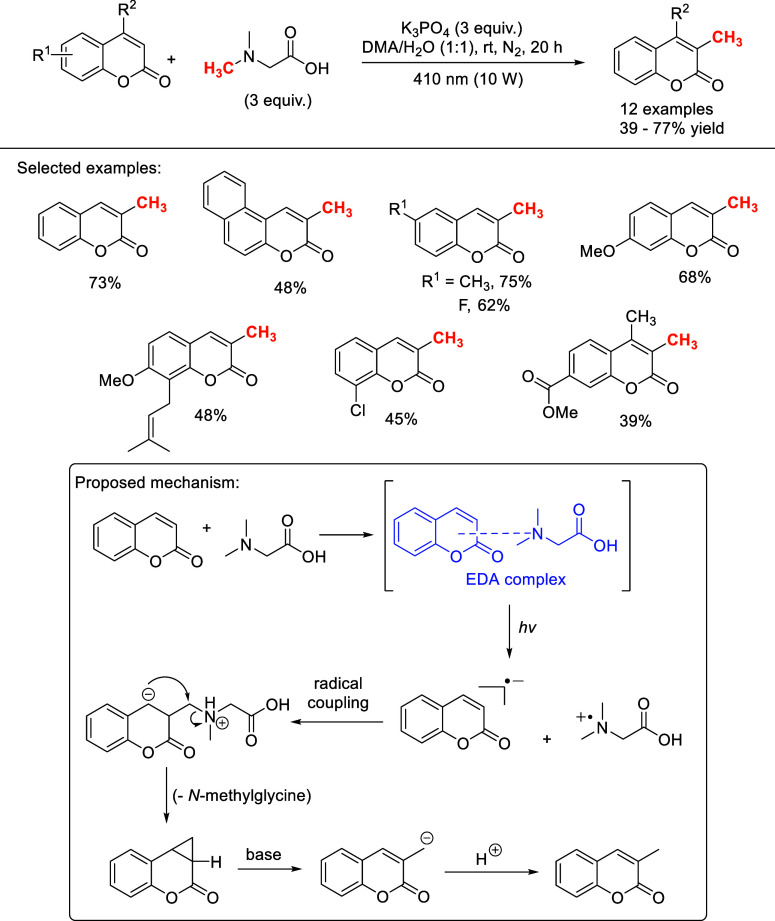
Regioselective Methylation of Heteroarenes Using Methylamines

### Alkyl Halides

Despite the inherent challenges associated with the direct activation of alkyl halides (the high bond dissociation energies of C–X bonds, particularly C–Cl and C–Br, and their relatively high reduction potentials), recent advances in photoredox strategies have overcome some of these limitations, enabling the efficient generation of alkyl radicals from unactivated precursors.^4,122,125^ In this context, the in situ generation of alkyl radicals from such substrates has arisen as a particularly attractive photoredox approach for the selective C3-alkylation of coumarins, offering mild reaction conditions and straightforward access to structurally diverse C3-functionalized derivatives.

In 2021, Wu and collaborators developed a direct radical alkylation methodology for coumarins using the commercially available 1,1,1-trifluoro-2-iodoethane as a source of the ^•^CH_2_CF_3_ radical.^153^ The optimized reaction conditions employed CF_3_CH_2_I (4 equiv) in the presence of the photocatalyst Ir­(ppy)_3_ (2 mol %) under blue LED irradiation (3 W) and basic conditions (K_2_CO_3_, 2 equiv) in DMSO (2 mL) at room temperature, affording the desired product in 89% yield for the model substrate (Scheme 29). This methodology demonstrated good functional group tolerance, providing the regioselective C3-alkylated products bearing alkyl (31–96%), alkoxy (41–94%), halogen (84–90%), and ester (42%) substituents at different positions of the coumarin aromatic ring. In the presence of a strong electron-withdrawing group (NO_2_), product was not detected. Regarding substituents at the C4 position, a methoxy group led to a moderate yield (51%), whereas a phenyl substituent afforded good yield (83–87%). Additionally, the biologically active molecules osthole and angelicin were successfully compatible, affording their trifluoroethylated analogues in 43% and 34% yields, respectively. Mechanistic studies were performed to investigate the reaction pathway. Radical detection experiments showed that the reaction was completely suppressed in the presence of two equivalents of TEMPO, resulting in the formation of the TEMPO-CH_2_CF_3_ adduct in 38% yield detected by GC–MS. 1,1-Diphenylethylene was also employed as a radical scavenger, reducing the reaction yield to 8% while its corresponding adduct was detected by GC–MS in 65% yield, strongly supporting the generation of the transient ^•^CH_2_CF_3_ radical. Furthermore, light on/off experiments revealed that product formation occurred only under continuous irradiation, ruling out a radical chain propagation pathway. Based on these observations, the proposed mechanism involves a single-electron transfer from the excited Ir­(ppy)_3_ photocatalyst to CF_3_CH_2_I, generating the ^•^CH_2_CF_3_ radical. This species undergoes regioselective addition to the C3 position of the coumarin to form a benzylic radical intermediate. Subsequently, a second single-electron transfer of this radical intermediate generates a carbocation while concurrently restoring the active Ir­(ppy)_3_ ground-state catalyst. Finally, deprotonation of the carbocation by the base furnishes the desired C3-trifluoroethylated coumarin.

**Scheme 29:**
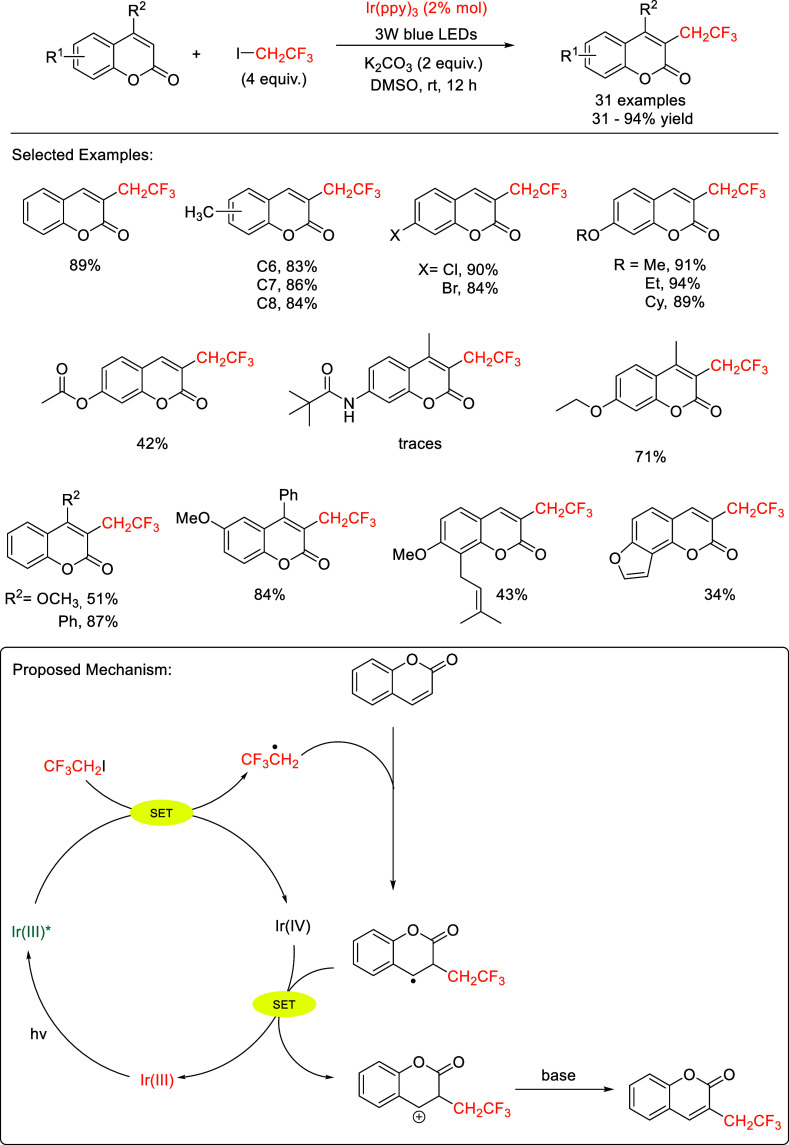
Iridium-Catalyzed Radical Alkylation of Coumarins Using Alkyl Iodine

Subsequently, a metal-free methodology for the C3-alkylation of coumarins using unactivated alkyl iodides was reported by Zhang and co-workers.^154^ The reaction was performed using alkyl iodides (3 equiv) in the presence of MTBD (1-methyl-1,3,4,6,7,8-hexahydro-2*H*-pyrimido­[1,2-a]­pyrimidine) (0.4 mmol) in DMSO under blue LED irradiation (6 W) at room temperature for 12 h (Scheme 30). Under these mild conditions, a variety of alkyl iodides were evaluated, including primary, secondary, and tertiary substrates, affording the corresponding 3-alkylated coumarins generally in moderate yields (40–80%). The methodology further enabled the incorporation of heterocyclic fragments such as oxetane, tetrahydrofuran, tetrahydropyran, and piperidine, providing the corresponding products in moderate yields (46–60%). The substrate scope for the coumarin core was also briefly investigated. Halogen substituents at the C7 position of the aromatic ring were well tolerated, with chloro- and fluoro-substituted coumarins delivering the alkylated products in 37% and 75% yields, respectively. Coumarins bearing electron-donating substituents such as methyl and methoxy, afforded the desired products in 44–65% yield. In addition, the bioactive coumarin derivative 7-(diethylamino)-4-methyl-2*H*-chromen-2-one proved to be compatible with the reaction conditions, giving the corresponding alkylated product in 37% yield. The practical applicability of the protocol was further demonstrated through gram-scale experiments, which provided the desired product in up to 75% yield. Mechanistic investigations suggest that the reaction proceeds through a radical pathway. Radical trapping experiments using TEMPO and BHT completely suppressed product formation, suggesting the involvement of radical intermediates. In addition, radical clock experiments employing cyclopropyl-containing iodides produced ring-opened products, further supporting the generation of transient alkyl radicals during the transformation. UV–vis absorption studies showed no evidence for the formation of an electron donor–acceptor (EDA) complex between the reaction components. Moreover, the measured quantum yield (Φ = 1.5) indicates that the process proceeds through a radical chain mechanism initiated by visible light rather than through a closed photocatalytic cycle. Further control experiments revealed that MTBD plays a key role in the initiation step, interacting with the alkyl iodide to generate the initial alkyl radical. This radical then adds to the electron-deficient C3 position of the coumarin, forming a carbon-centered radical intermediate. Subsequent deprotonation by MTBD generates a radical anion intermediate, which acts as a strong reducing species capable of reducing another molecule of alkyl iodide through single-electron transfer. This final step generates another equivalent of the alkyl radical while forming the desired alkylated product, thereby efficiently sustaining the radical chain process.

**Scheme 30:**
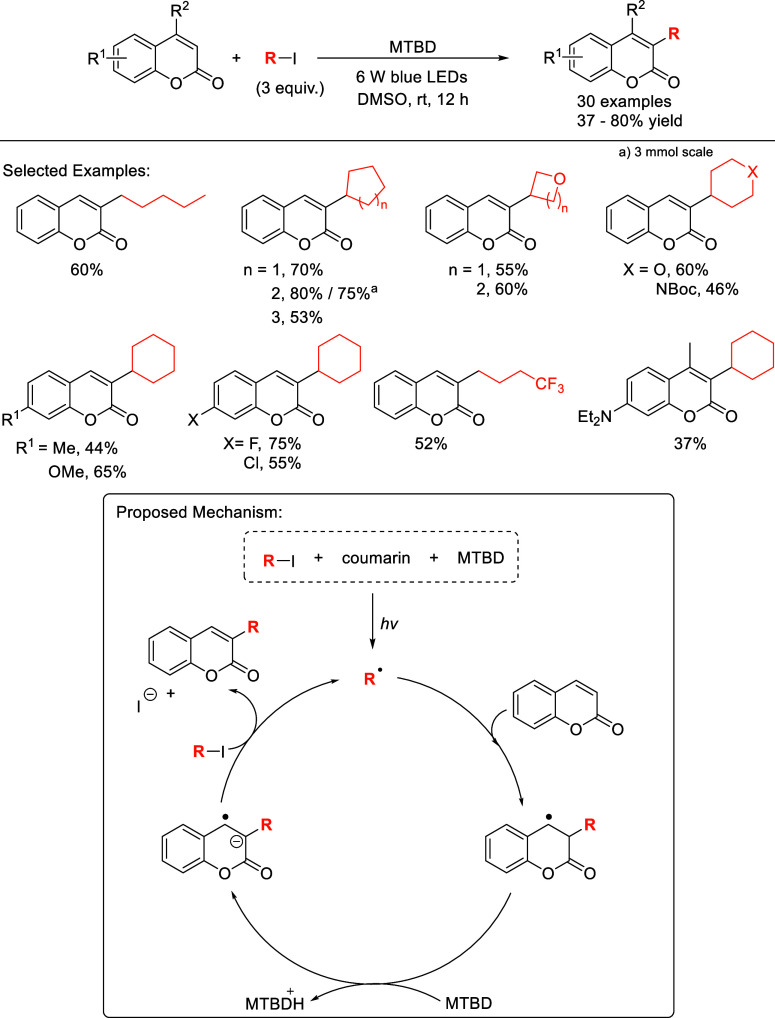
Metal-Free Radical Alkylation of Coumarins Using Alkyl Iodine as Radical Source

In 2024, He and co-workers reported a radical cross-coupling strategy between α-trifluoromethylated alkyl bromides and coumarins enabled by halogen-bond (XB) activation (Scheme 31).^155^ Reaction optimization identified DMSO as the optimal solvent and DMAP as dual-functional halogen-bond acceptor and base under visible-light irradiation (purple LED, 410–415 nm, 12 h). To maximize the reaction efficiency, the coumarin substrate was employed in excess (10 equiv) affording the desired product in 82% yield. Importantly, the unreacted coumarin could be efficiently recovered after the process (76% recovery). Screening of different electron donors revealed that DMAP was uniquely effective, highlighting the crucial role of halogen-bond formation in the activation process. This strategy exploits the ability of the CF_3_ group to strengthen halogen-bond interactions while weakening the adjacent C–Br bond, as supported by both experimental observations and DFT calculations, thereby enabling efficient radical generation under mild conditions. The scope of the reaction was investigated using a variety of α-CF_3_ alkyl bromides. A broad range of substrates bearing electron-donating, electron-neutral, and electron-withdrawing substituents were well tolerated, delivering the corresponding products in moderate to high yields (35 examples, up to 85% yield). Notably, the protocol could be performed on gram-scale without any significant loss of efficiency (75% yield). The applicability of the method was further demonstrated with a structurally diverse set of coumarins, including biologically active derivatives. In addition, the reaction was successfully extended to other heterocycles, such as quinolinones, pyridones, indoles, and imidazopyridines, highlighting the versatility of the transformation and its potential for late-stage functionalization of bioactive scaffolds. Control experiments demonstrated that both DMAP and light irradiation were essential for the transformation. The presence of the radical scavenger TEMPO completely suppressed product formation, indicating the involvement of radical intermediates. Furthermore, replacing the CF_3_ substituent with less electron-withdrawing groups, such as CH_3_ or CF_2_H, resulted in no detectable product, underscoring the crucial role of the CF_3_ group in promoting C–Br bond activation. Light on/off experiments further confirmed the necessity of continuous irradiation, ruling out a radical chain propagation mechanism. Based on these observations, the authors proposed a mechanism in which the α-CF_3_ alkyl bromide initially assembles into a halogen-bond complex with DMAP. Upon visible-light irradiation, this interaction facilitates single-electron transfer and homolytic cleavage of the C–Br bond to generate an alkyl radical. The resulting radical adds to the C3 position of the coumarin, forming a radical intermediate that is subsequently undergoes subsequent oxidation to a carbocation, followed by deprotonation to furnish the final C3-alkylated coumarin. The same research group further applied this halogen-bond activation strategy to other electron-deficient radical precursors for coumarin alkylation, thereby significantly expanding the substrate scope and enhancing the synthetic utility of the methodology.^156–158^

**Scheme 31:**
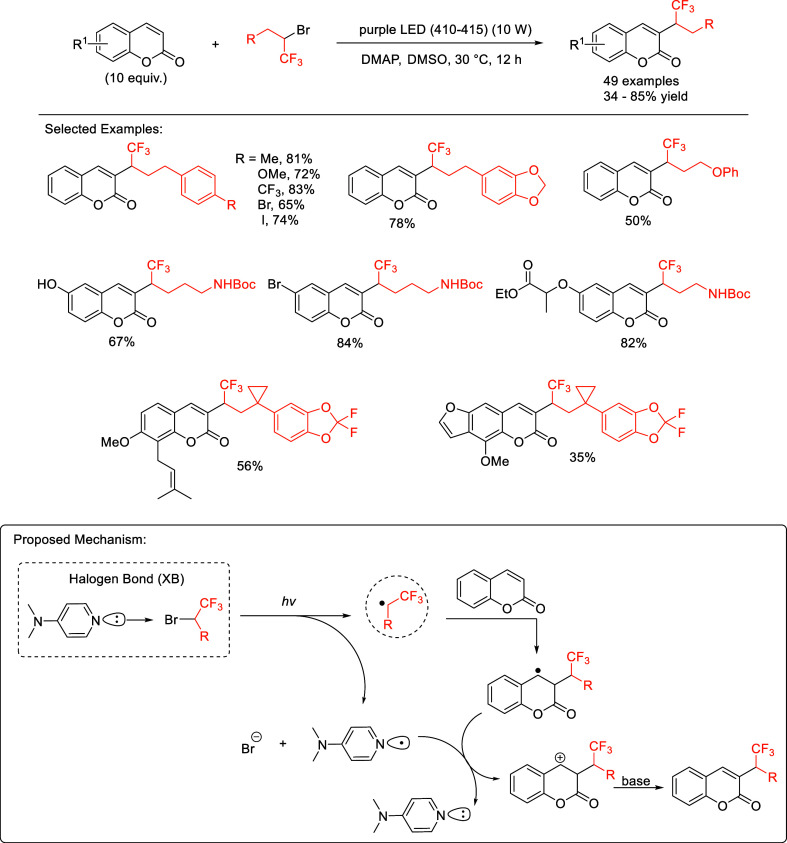
Halogen-Bond Activation Strategy to Promote Alkylation of Coumarins

Another important metal-free photoredox strategy for direct C3-alkylation of coumarins using alkyl halides as radical precursors was developed by Tang and co-workers, providing a practical approach to access structurally diverse coumarin derivatives under mild reaction conditions (Scheme 32).^159^ The transformation was performed using 7-diethylamino-4-methylcoumarin as the model substrate, NaHCO_3_ as the base, and DMSO as the solvent under irradiation at 405 or 365 nm, notably avoiding the need for transition-metal catalysts or external photocatalysts. Although the method was initially optimized using activated alkyl bromides as radical sources, it was subsequently extended to a variety of alkyl halides, including unactivated alkyl iodides, affording the desired regioselective C3-alkylated products in moderate to good yields (36–81%). The scope of the coumarin substrates was also explored. In the case of reactions employing unactivated alkyl iodides, coumarins bearing electron-donating groups, such as methoxy and methyl substituents at the C7 position of the aromatic ring, furnished the corresponding products in 41% and 52% yield, respectively. Mechanistic investigations were conducted to elucidate the reaction pathway. UV–Vis spectroscopic analysis revealed no evidence for the formation of an electron donor–acceptor (EDA) complex. In addition, radical trapping experiments using TEMPO suggested the involvement of radical intermediates. The formation of the corresponding TEMPO-alkyl radical adduct was successfully detected by GC–MS and quantified by ^19^F NMR spectroscopy in 71% yield. Further spectroscopic and control experiments indicated that coumarin acts not only as the substrate but also as the photoactive species responsible for initiating the reaction. Upon visible-light irradiation, coumarin (Cou) is promoted to its singlet excited state (*S*_1_), which undergoes single-electron transfer with the alkyl halide to generate the alkyl radical. This transient radical then selectively adds to the electron-deficient C3 position of the coumarin framework, forming a new carbon–carbon bond and generating a benzylic radical intermediate. Subsequent oxidation and deprotonation restore the aromatic system, ultimately affording the C3-alkylated coumarin product.

**Scheme 32:**
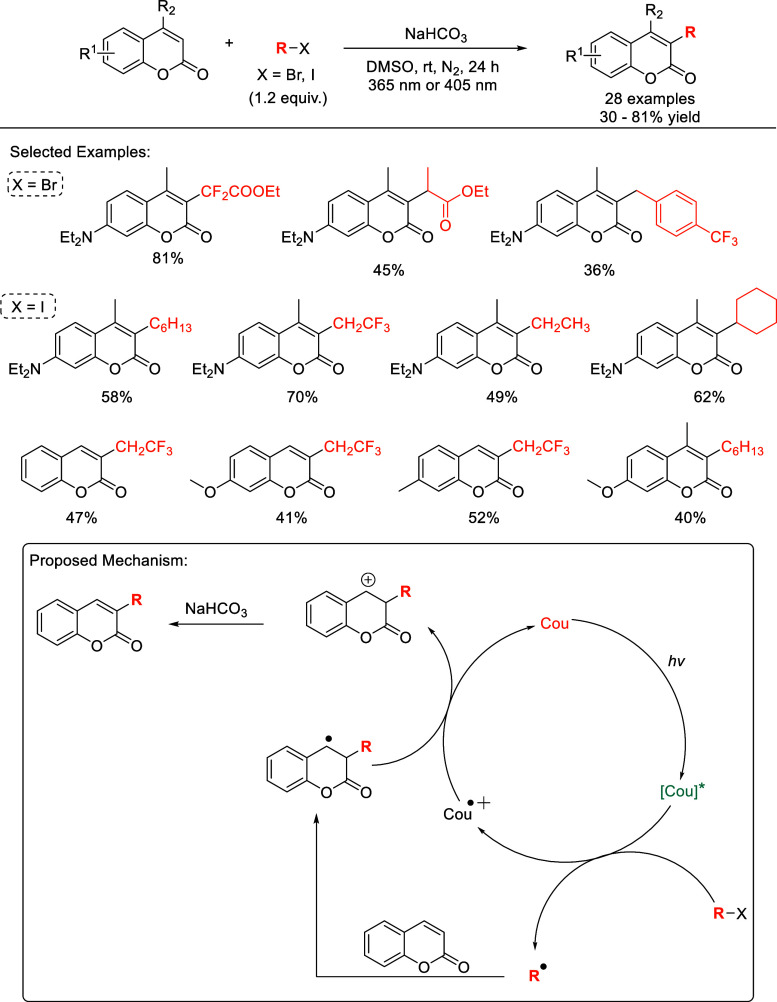
Metal-Free Radical Alkylation of Coumarins Using Alkyl Halides

When evaluating these recent contributions, a clear trend regarding the reactivity and limitations of the radical precursors becomes evident. For simple alkylations, existing methodologies typically rely on alkyl iodides, often requiring a large excess of reagent to achieve high chemical efficiency. In contrast, the use of alkyl bromides remains limited to activated substrates or highly specialized electronic systems. Consequently, despite the promise of these strategies, significant advances are still needed to develop protocols that effectively employ unactivated and readily available halides (X = Br, Cl) with lower substrate loadings.

## Conclusion

Over the past decade, radical-mediated strategies have emerged as powerful and versatile tools for the direct C3 functionalization of coumarins. By enabling carbon–carbon bonds formation under relatively mild conditions and with broad functional group tolerance, these approaches have overcome several limitations associated with traditional methodologies that rely on specific prefunctionalized substrates and multistep sequences. Both thermal and photoredox strategies have demonstrated significant potential, providing complementary platforms for the generation of alkyl radicals from a diverse range of precursors.

Thermal approaches, often based on peroxide activation or transition-metal catalysis, offer operational simplicity and access to a wide range of radical sources, including alkanes, ethers, and amines. However, these methods frequently require elevated temperatures, excess reagents, and strong oxidants, which may compromise functional group compatibility and sustainability. In contrast, photoredox strategies enable radical generation under significantly milder conditions, often at room temperature and using visible light as a traceless energy input. These methodologies have expanded the scope of accessible radical precursors and improved selectivity, although challenges related to catalyst cost, scalability, and substrate scope remain.

Despite notable progress, several important limitations persist in the field of C3 radical functionalization of coumarins. Most reported methodologies are still biased toward stabilized or activated radical precursors. The efficient incorporation of nonstabilized primary alkyl radicals, as well as more complex and functionalized motifs, remains comparatively underexplored. Furthermore, the scope of coumarin substrates is often restricted, with reduced reactivity observed for electron-deficient systems or sterically hindered derivatives, particularly those bearing substituents at the C4 position.

Looking forward, several key directions can be identified for the future development of this field. First, the design of new catalytic systems capable of promoting the generation and selective addition of nonstabilized and structurally diverse radical species will be crucial to expand the chemical space accessible through C3 functionalization. Additionally, the exploration of vinyl and alkynyl radical precursors for direct C3 functionalization remains largely unexplored and could open new avenues for the synthesis of structurally complex and conjugated coumarin derivatives. From a sustainability perspective, future efforts should also focus on the development of greener and more practical protocols, including metal-free systems, the use of earth-abundant catalysts, and the implementation of electrochemical strategies that minimize waste generation and improve energy efficiency.

In summary, although significant progress has been achieved, the field of C3 radical functionalization of coumarins remains rich with opportunities. Continued advances in reaction design, mechanistic understanding, and catalyst development are expected to further expand the scope and synthetic utility of these transformations, consolidating their role as powerful strategies in modern synthetic chemistry.
